# Contrasting response of rainfall extremes to increase in surface air and dewpoint temperatures at urban locations in India

**DOI:** 10.1038/s41598-017-01306-1

**Published:** 2017-04-27

**Authors:** Haider Ali, Vimal Mishra

**Affiliations:** Civil Engineering, Indian Institute of Technology (IIT) Gandhinagar, Gujarat, India

## Abstract

Rainfall extremes are projected to increase under the warming climate. The Clausius-Clapeyron (C-C) relationship provides a physical basis to understand the sensitivity of rainfall extremes in response to warming, however, relationships between rainfall extremes and air temperature over tropical regions remain uncertain. Here, using station based observations and remotely sensed rainfall, we show that at a majority of urban locations, rainfall extremes show a negative scaling relationship against surface air temperature (SAT) in India. The negative relationship between rainfall extremes and SAT in India can be attributed to cooling (SAT) due to the monsoon season rain events in India, suggesting that SAT alone is not a good predictor of rainfall extremes in India. In contrast, a strong (higher than C-C rate) positive relationship between rainfall extremes and dew point (DPT) and tropospheric temperature (T850) is shown for most of the stations, which was previously unexplored. Subsequently, DPT and T850 were used as covariates for non-stationary daily design storms. Higher magnitude design storms were obtained under the assumption of a non-stationary climate. The contrasting relationship between rainfall extremes with SAT and DPT has implications for understanding the changes in rainfall extremes in India under the projected climate.

## Introduction

Extreme rainfall events may lead to flooding which disrupts urban transportation and often cause damage to infrastructure. The intensity and frequency of extreme rainfall events are projected to increase under climate warming^1–8^, which is supported by observations as well as climate model simulations. The Clausius-Clapeyron (C-C) relationship can be used as a physical basis to evaluate the sensitivity of rainfall extremes against changes in air temperature^9–13^. The water holding capacity of atmosphere increases by approximately 6–7%/K increase in air temperature according to the C-C relationship. Furthermore, atmospheric humidity increases with the same rate provided relative humidity remain constant^14–16^.

Rainfall extremes may show higher scaling than suggested by the C-C relationship, which may be due to convective nature of rainfall or excess latent heat released during intense rainfall^17, 18^. The precipitation-temperature relationship may vary with intensity and temporal resolution of rainfall extremes^15, 19, 20^. For instance, higher scaling rates for sub-daily rainfall extremes than daily extremes are reported in previous studies^10, 21, 22^. The precipitation-temperature relationship can also be affected by the other factors such as duration and type of a storm event^23–25^, temperature^26^, season and the geographical location where the storm occurs^27, 28^. For instance, Wasko *et al*.^24^ stated that the scaling decreases with an increase in the frequency and duration of a storm event. Furthermore, convective events are more sensitive to temperature^29^ and show higher scaling than stratiform rainfall^30^, which is supported by the findings of Moseley *et al*.^31^. Moseley *et al*.^31^ showed that an increase in temperature intensifies cloud-cloud interaction which leads to stronger precipitation.

In India, major rainfall occurs through convective storms which have high-temperature dependency^32^. Therefore, scaling of rainfall extremes with surface air temperature (SAT) may not be a good indicator of climatic change^33^. Moreover, high SAT during the pre-monsoon (March–May) season most often get cooled down due to rain events, which results in a negative relationship between SAT and rainfall during the monsoon season. For example, Vittal *et al*.^34^ used the relationship between extreme rainfall events with 2 m SAT over India and found negative scaling rates for most of the regions, which are primarily due to the dominant negative relationship between SAT and monsoon season rainfall.

Since diurnal variations in SAT in response to rainfall may provide improper scaling rates, a relationship of rainfall extremes with T850 (or in the upper troposphere, temperature at 850 hPa), which is at a height sufficient enough to avoid these variations may be robust^10^. Furthermore, the relationship between rainfall and humidity may be a good predictor to analyse rainfall extremes under the warmer climate^15^. Trenberth *et al*.^16^ hypothesized that rainfall intensity increases at about the same rate as atmospheric moisture and moisture availability becomes the dominant driver of extreme precipitation at higher temperatures (299 K)^35^. Relative humidity and dewpoint temperature (DPT) are related as DPT corresponds to the air temperature at which the air is completely saturated with water (i.e. Relative humidity is 100%). Therefore, Lenderink and Van Meijgaard^33^ considered DPT as a direct measure of atmospheric humidity and showed that in tropical regions rainfall extremes display a better relationship with DPT than SAT.

Urban areas in India face frequent flooding caused by extreme rainfall events. Large built-up and impervious fraction in urban areas lead to increased sensible heat, which in turn, can increase temperature by 2–10 °C than the surrounding non-urban areas^35^. Urban stormwater infrastructures designs are based on intensity duration frequency (IDF) curves, which are usually developed using an annual maximum rainfall series assuming stationary conditions in India^36, 37^. However, in the present scenario, annual maximum rainfall cannot be assumed to have a time-invariant probability density function^36, 38, 39^. For instance, Cheng and AghaKouchak^40^ reported significant differences in stationary and nonstationary intensity duration frequency (IDF) curves estimated for a shorter duration at a few stations in the USA. Similarly, Verdon-Kidd^41^ showed the potential role of non-stationarity conditions on IDF curves in Australia.

Despite the need of an improved understanding of rainfall extremes in urban areas, efforts to evaluate the scaling relationship between rainfall extremes and SAT, T850, and DPT in India have been limited. This may be because of a lack of station based observations for rainfall, SAT, and DPT for urban areas. Using station data from the Global Summary of the Day (GSOD) and other gridded datasets, we provide an assessment of the sensitivity of precipitation extremes in urban areas over India against SAT, DPT, and T850. Moreover, the relationship of daily and sub-daily rainfall extremes with T850 and DPT may help to improve our understanding of precipitation extremes, which might have strong implications for urban stormwater designs, especially under non-stationary climate conditions. Here, we aim to address the following questions: (1) how sensitive are daily and sub-daily rainfall extremes to SAT, T850, and DPT in major urban areas in India? and (2) to what extent nonstationary atmospheric conditions based on DPT and T850 as covariates influence urban stormwater design estimates in India?

## Results and Discussion

Most of the GSOD observation stations are located in urban areas (or at nearby airports) and their distance from the city center varies between 1 and 13 km (Supplemental Table S1). Therefore, the selected stations can provide information of rainfall extremes in urban locations especially in the absence of observation stations within urban areas. We acknowledge that these stations may not truly represent urban micro-climate or the factors that affect urban meteorology, for which, a larger number of stations within urban areas will be needed, and are currently unavailable in India. Notwithstanding this limitation, the station based GSOD data can provide valuable information about the relationship between rainfall extremes and temperature at urban locations.

### Scaling of rainfall extremes with surface air temperature (SAT)

Rainfall data were obtained from the observed station based daily GSOD (period: 1979–2015), Tropical Rainfall Measurement Mission Multi-satellite Precipitation Analysis (TMPA) 3B42v7 rainfall product (TRMM; daily and 3-hourly; period: 1998–2015), and Climate Hazards Group Infra-Red Precipitation with Station data (CHIRPS; daily; period: 1981–2015). Station based observations for SAT were obtained from GSOD data for 1979–2015. Since rainfall datasets are available at different spatial resolutions, we applied areal reduction factors^10^ to bring all the datasets to point scale (consistent with GSOD data). Using quantile regression^28, 41–44^ (QR), we estimated regression slopes (dR95/K, %) as change (%) in the 95th percentile of rainfall (magnitude greater or equal to 1 mm) with respect to change in daily mean SAT. To check if the regression slopes obtained from the QR method are robust, we estimated regression slopes also using binning techniques (BT)^10, 15, 21, 33, 45^. We distributed rainfall data and their corresponding daily mean SAT into 20 bins of the same size that were sorted from the lowest to highest daily mean SAT. Then the 95th percentile of rainfall (R95) and median of daily SAT for each bin was estimated and linear regression of logarithm of R95 and SAT was performed. Then regression slopes were estimated using the regression relationship between the lowest daily mean SAT (mean SAT of the first bin) and the daily mean SAT at the peak point temperature (SAT_R95_).

**Figure 1:**
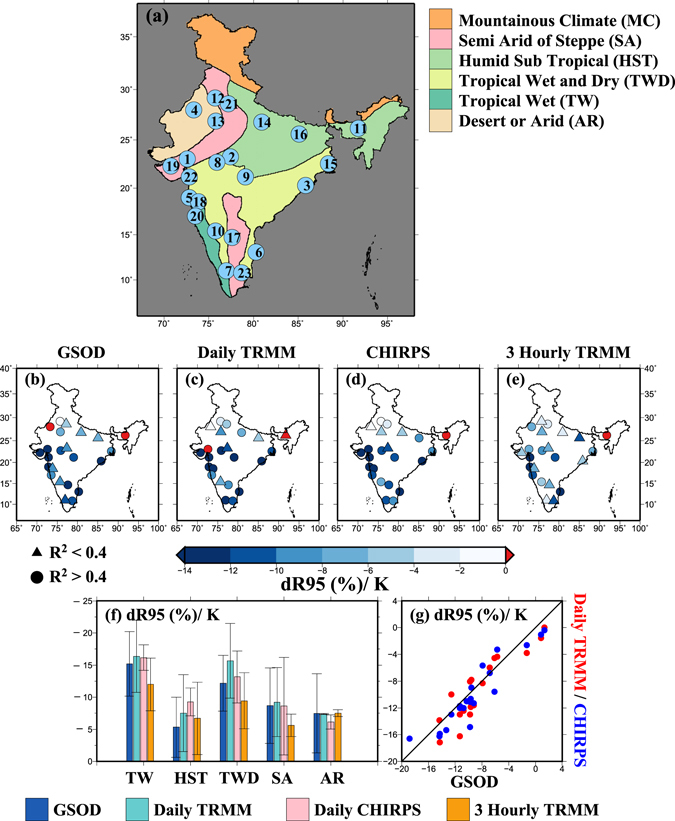
(**a**) Location of selected urban areas and different climatic zones in India, (**b**–**e**) regression slopes (dR95/K, %) obtained from daily GSOD, daily TRMM, daily CHIRPS and 3-hourly TRMM data, respectively with surface air temperature (SAT) using quantile regression (QR) at the 95th percentile for 23 urban areas across India, (**f**) regression slopes (dR95/K) from daily GSOD (blue), daily TRMM (cyan), daily CHIRPS (pink) and 3-hourly TRMM (orange) data for different climatic zones respectively where bars denote mean values and whiskers show standard deviations, (**g**) agreement in scaling results between GSOD and Daily TRMM (red) and GSOD and CHIRPS (blue), pooled for all 23 urban areas. The figure was developed using the Generic Mapping Tools (GMT, https://www.soest.hawaii. edu/gmt/).

We find that regression slopes between extreme rainfall and daily mean SAT are negative for most of the locations for all the rainfall datasets (Fig. 1b–e). For instance, regression slopes between daily rainfall and mean SAT from GSOD data are negative for 21 locations out of total 23. Moreover, we find a relatively stronger negative relationship between daily and sub-daily rainfall extremes and mean SAT for the locations in the southern India (Fig. 1). The negative relationship between rainfall extremes and daily mean SAT^46^ can be attributed to rainfall-induced cooling in surface air temperature in India^47^ (Fig. S7). The pre-monsoon season (March to May) is the warmest in India and surface air temperature declines after rain events, which is clearly reflected by the strong negative relationship as shown by our results (Fig. S7). Moreover, we estimated scaling relationships between extreme rainfall and daily maximum SAT^45^, and daily mean SAT (1 and 3 days prior to rainfall) to further understand the role of SAT on rainfall extremes (Fig. S8). The relationship between rainfall extremes and daily maximum SAT was largely negative at most of the locations^45^ (Fig. S8b). However, daily mean SAT for 1 and 3 days prior to the rain event, the relationship was positive for a few stations indicating the role of surface temperature prior to rain event on the scaling relationship.

Differences in regression slopes based on rainfall duration and climatic zones show relatively less negative regression slopes for 3-hourly rainfall extremes from TRMM (Fig. 1f). Moreover, locations in the tropical wet (TW) and tropical wet and dry (TWD) show higher negative regression slopes than that of the other climatic zones (Fig. 1f). We observed a decline in R95 with an increase in SAT, which shows a negative relationship between rainfall and SAT (Fig. S8). Regression slopes obtained from QR are relatively more negative than those obtained from the BT since regression slopes are estimated only up to peak point temperature in the later method (Fig. S6). We also notice that the peak point temperature may vary (up to 2.5 K) within the climatic zone, which might be related to the geographical and climatic settings of the urban areas or location of the GSOD stations (Fig. S6j). Robustness in scaling results from the both methods (QR and BT) can be seen in Fig. S9.

The negative relationship between rainfall extremes and daily mean SAT for most of the locations in India provide some important insights. For instance, Vittal *et al*.^34^ reported that the C-C relationship is valid for the mid-latitude region; however, the response of rainfall extremes towards an increased warming over the tropical region is debatable. The temperature relationship of extreme precipitation intensity on a global scale remains unclear, however, extreme precipitation intensity in response to higher temperature increases at mid-latitudes while declining over the tropics^22^. Our findings are consistent with Maeda *et al*.^48^ as they showed a negative relationship between the magnitude of precipitation and higher temperature at daily time scales. Here we argue that despite the negative relationship between extreme precipitation and air temperature in India, surface temperature alone may not be sufficient to understand the changes of precipitation extreme under the warming climate^48^. Over the monsoon regions in the tropics, this negative relationship largely reflects the response of surface air temperature to rainfall rather than a cause. However, a robust relationship between daily mean SAT and rainfall extremes can be obtained using mean SAT prior to rain events.

### Scaling rainfall extremes with air temperature at 850 hPa (T850)

**Figure 2:**
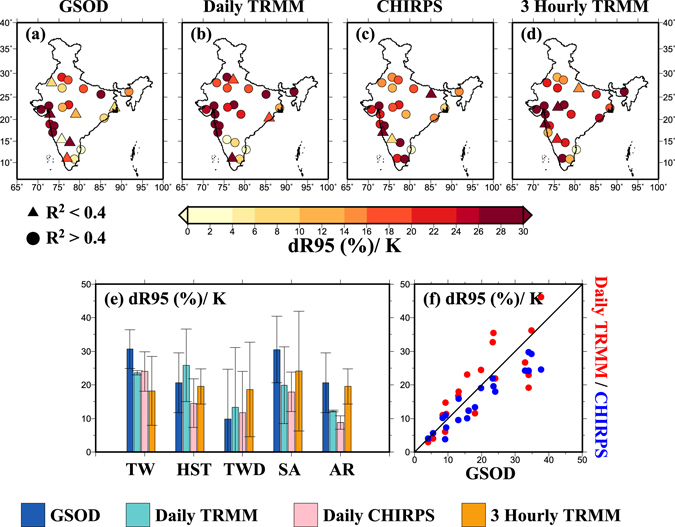
(**a**–**d**) Regression slopes (dR95/K, %) obtained from daily GSOD, daily TRMM, daily CHIRPS and 3-hourly TRMM data, respectively with air temperature at 850 hPa (T850) using quantile regression (QR) at the 95th percentile for 23 urban areas across India, (**e**) regression slopes (dR95/K) from daily GSOD (blue), daily TRMM (cyan), daily CHIRPS (pink) and 3-hourly TRMM (orange) data for different climatic zones respectively where bars denote mean values and whiskers show standard deviations, (**f**) agreement in scaling results between GSOD and Daily TRMM (red) and GSOD and CHIRPS (blue), pooled for all 23 urban areas. The figure was developed using the Generic Mapping Tools (GMT, https://www.soest.hawaii.edu/gmt/).

Since SAT during the monsoon season is driven by the local rainfall event in India, we established a scaling relationship between daily and sub-daily rainfall extremes and tropospheric air temperature at 850 hPa (T850) as increasing tropospheric temperature can lead to higher precipitation intensities^49–51^. Similar to SAT, regression slopes from daily and 3-hourly rainfall extremes and daily mean T850 were obtained using QR and BT methods for the 23 locations in India (Fig. 2). Daily rainfall extremes from GSOD, TRMM, and CHIRPS data showed regression slopes higher than 7%/K (super scaling of C-C) at 16 out of 23 locations. We found a consistent super-scaling C-C relationship across the datasets for daily rainfall extremes as well as for 3-hourly rainfall extremes from TRMM (Fig. 2a–d). However, Chennai showed a negative (−6%) regression slope between rainfall extremes and daily mean T850, which can be attributed to the seasonal difference in the occurrence of rainfall extremes. For instance, in the southern peninsula, rainfall occurs during the winter (November to January) season primarily due to the northwest monsoon. Therefore, in south India, the relationship between T850 and rainfall may not be as strongly positive as obtained in the north India, where most of the extreme rainfall events occur during the summer monsoon (June to September).

We find that a majority of the locations show a good relationship between rainfall extremes and daily mean T850 with R^2^ values greater than 0.4. Moreover, 3-hourly rainfall extremes from TRMM showed a regression slope greater than 7% (median 20%) for 19 out of 23 urban areas indicating a higher sensitivity of rainfall extremes at shorter durations. We find a variation in the regression slopes based on the datasets and climatic regions (Fig. 2e) that can be associated with data length and different methods that are used to process the gridded datasets (TRMM and CHIRPS)^52–57^. However, at a daily scale, the relationship obtained from the gridded datasets shows a good agreement with that obtained using station data from GSOD (Fig. 2f). Moreover, regression slopes obtained from QR were found to be consistent with BT (Figs S9 and S10). We find an increase in rainfall intensity with T850 for all the stations pooled for the same climatic zone (Fig. S9).

Gridded satellite (TRMM and CHIRPS) datasets provide sparse rain networks for sub-regional applications, however, due to seasonal and climatic dependence; they may have uncertainties at the local scale^58^. For example, TRMM data may miss finer details on local rain as compared to IMD gridded data over India, however, show a higher correlation with rain-gauge-based estimates as compared to GPCP (Global Precipitation Climatology Center) and GSMaP (Global Satellite Mapping of Precipitation)^59, 60^. Nair *et al*.^61^ also compared gridded TRMM data with rain-gauge observations over western ghats in India and found that TRMM give accurate rainfall estimates in regions of moderate rainfall and inaccurate estimates (overestimate) in the region of sharp rainfall gradient. Similarly, CHIRPS showed a higher correlation (>0.75) with wet season GPCC precipitation in India than TRMM, CFS, and ECMWF^53^. Gridded precipitation products have been widely used to understand the variability of precipitation extremes in urban areas^32, 62–64^ and may have uncertainties due to retrieval and post-processing methods^65–67^. However, scaling relationship obtained from station and satellite-based data sets for urban locations in India demonstrated robustness in our results.

Since tropospheric air temperature was obtained from the reanalysis datasets, we evaluated the robustness of our results by comparing the scaling relationship obtained using T850 from the three reanalysis datasets (ERA-Interim, MERRA 2, and CFSR). We find a consistent relationship between rainfall extremes and daily mean T850 from all the three reanalysis products and for both QR and BT methods (Figs S11–S14). We also developed mean sea level pressure (SLP) and T850 composites to understand their variability during the extreme rainfall events at the selected urban locations suggesting a role of tropospheric temperature anomaly on rainfall extremes (see Supplemental text and Figs S2–S4).

### Scaling of rainfall extremes with daily dewpoint temperature (DPT)

**Figure 3:**
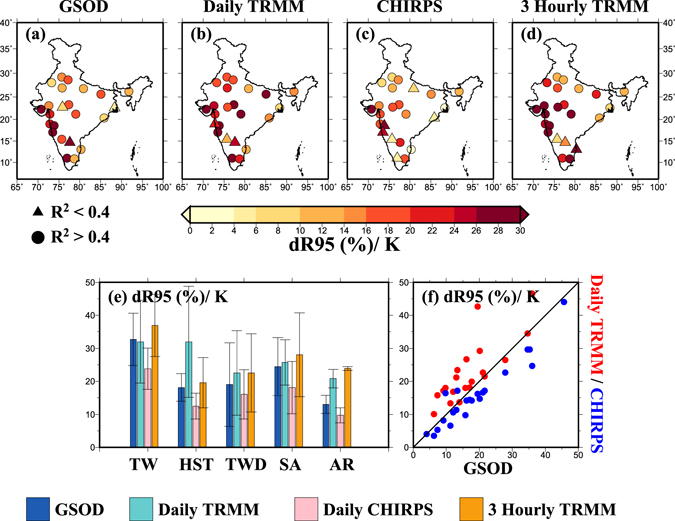
(**a**–**d**) Regression slopes (dR95/K, %) obtained from daily GSOD, daily TRMM, daily CHIRPS and 3-hourly TRMM data, respectively with dewpoint temperature (DPT) using quantile regression (QR) at the 95th percentile for 23 urban areas across India, (**e**) regression slopes (dR95/K) from daily GSOD (blue), daily TRMM (cyan), daily CHIRPS (pink) and 3-hourly TRMM (orange) data for different climatic zones respectively where bars denote mean values and whiskers show standard deviations, (**f**) agreement in scaling results between GSOD and Daily TRMM (red) and GSOD and CHIRPS (blue), pooled for all 23 urban areas. The figure was developed using the Generic Mapping Tools (GMT, https://www.soest.hawaii.edu/gmt/).

Since the relationship between rainfall extremes and humidity may be a good predictor to analyse rainfall extremes under the warmer climate, we considered dewpoint temperature as a measure of absolute humidity^15, 33^. We find that regression slopes obtained using QR are greater than the C-C rate (~7%) for most of the daily rainfall datasets and at most of the urban locations (Fig. 3a–d, Fig. S11). Only 4 (Bhubaneshwar, Bikaner, Indore, and Kolkata) out of total 23 locations showed regression slopes lesser than the C-C rate. Moreover, 3-hourly rainfall extremes from TRMM showed a super C-C relationship (median 22%) for all 23 locations. 3-hourly rainfall extremes from TRMM showed higher regression slopes than that of daily rainfall extremes for most of the climatic zones (Fig. 3e). We find a good agreement between regression slopes obtained from the gridded datasets and with those obtained from the station data from GSOD (Fig. 3f) and for both QR and BT methods (Figs S15 and S9i–l). We notice that 3-hourly data from TRMM provide valuable information on the sensitivity of rainfall extremes against DPT, however, a long-term station data with sub-daily durations are desirable for robust estimation of the scaling relationship.

**Figure 4:**
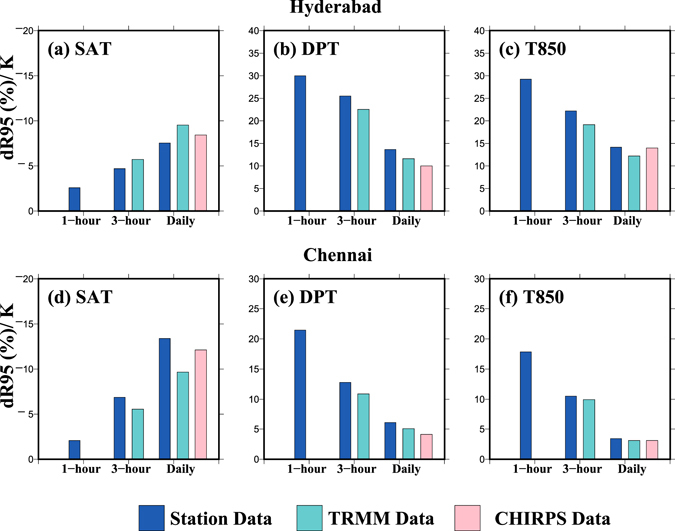
(**a**–**c**) Regression slopes (dR95/K, %) obtained using rainfall from station (blue; for 1979–2013), TRMM (cyan; for 1998–2013) and CHIRPS (pink; for 1979–2013) data with (**a**) SAT, (**b**) DPT, and (**c**) T850, respectively for 1-hour, 3-hour and daily durations for Hyderabad using quantile regression (QR) at the 95th percentile, (**d**–**f**) same as (**a**–**c**) but for Chennai for period 2008–2013 for all the datasets. The figure was developed using the Generic Mapping Tools (GMT, https://www.soest.hawaii.edu/gmt/).

To further evaluate the robustness of the scaling relationship,we obtained observed hourly rainfall data for two stations: Hyderabad (1979–2013) and Chennai (2008–2013) (Fig. 4). For both stations with hourly rainfall observations, we performed the quantile regression (QR) on 1 hour, 3 hour, and daily rainfall durations at 95^th^ percentile. Hourly rainfall extremes show a negative scaling relationship with SAT while positive scaling relationship with DPT and T850 (Fig. 4). Moreover, the regression slope between hourly extreme rainfall and DPT/T850 was substantially larger (super C-C) than the slope obtained for 3-hourly and daily rainfall durations, which are consistent with previous studies^15, 21^. We also find that our scaling results obtained from TRMM and CHIRPS for 3-hourly and daily durations are consistent with station based observations (Fig. 4).

We observed higher regression slopes than the C-C rate for daily and 3-hourly rainfall extremes against DPT and T850 for most urban locations, which is consistent with the previous studies^15, 33, 68^. Hardwick-Jones *et al*.^45^ showed that the scaling relationship increases with temperature till temperature reaches at 25 °C and declines afterward. Similar variation in rainfall extremes with air temperature was observed in the tropical regions by Utsumi *et al*.^22^. Rainfall extremes in India generally occur at higher temperatures (more than 25 °C), a negative scaling relationship between rainfall extremes and daily mean SAT is observed for most of the locations using the station and gridded datasets. This negative relationship between rainfall extremes and daily mean SAT in India may not be sufficient to understand the nature of rainfall extremes under the warming climate. The relationship between daily/sub-daily rainfall extremes and dew point temperature is more robust and can be used to understand the changes in rainfall intensity under the warming climate^15, 33, 69^ in contrast to the findings based on the relationship with SAT as reported in Vittal *et al*.^34^.

It remains unclear if the super C-C relationship exhibited by daily and sub-daily extremes in India is linked with convective nature of rainfall. For instance, Haerter and Berg^17^ argued that due to a shift from stratified to convective precipitation, precipitation extremes can show the super C-C relationship. Moreover, Pall *et al*.^11^ also reported the super C-C feedback on convective precipitation due to the release of latent heat during rain events. To evaluate if the majority of rainfall extremes over India occur due to convective precipitation, we used convective rainfall data (CON_RAIN) from ERA-Interim reanalysis and found that convective rainfall contributes around 80% of total rainfall (TOT_RAIN; obtained from ERA-Interim) for most of the locations for 1979–2015 (Fig. S16a). Moreover, after determining that a majority of rainfall extremes are of convective in nature, we established the relationship between convective rainfall extremes and DPT/T850. Our results show that the scaling rates obtained using both the methods (QR and BT) for CON_RAIN (from ERA-Interim) and TOT_RAIN (from GSOD and ERA-Interim) are similar indicating that most of the extreme rainfall events are driven by convective storms (Figs S16 and S17). The reason for higher scaling rate of convective rainfall against DPT/T850 can be attributed to the higher sensitivity of convective precipitation to temperature^30, 31^.

For 3-hourly rainfall extremes, we notice that regression slopes are higher than for daily extremes, which is consistent with the findings of Miao *et al*.^70^ who reported that sub-daily rainfall extremes increasing at three times the C-C rate with air temperature over the tropical regions of China. Scaling of rainfall extremes with DPT provides more robust results for the sensitivity of rainfall extremes under the warming climate in India, where convection is major rainfall causing mechanism^15, 69–72^. Our results show that SAT may not be the most appropriate to evaluate temperature sensitivity of rainfall extremes under the warming climate in India.

### Scaling of rainfall extremes with T850 and DPT in urban and non-urban areas

**Figure 5:**
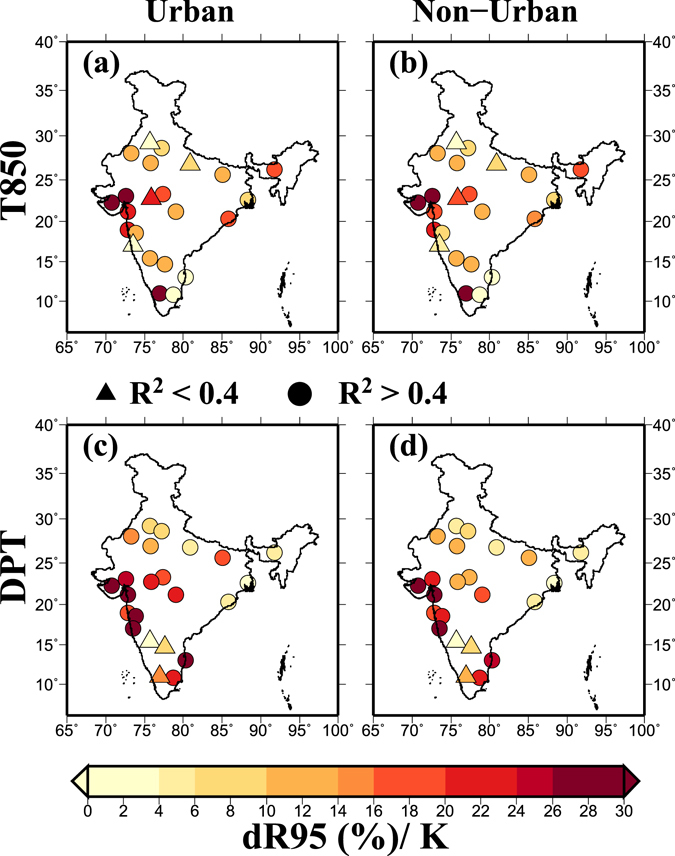
(**a**,**b**) Regression slopes (dR95/K, %) obtained from 3-hourly TRMM data using quantile regression (QR) at the 95th percentile for urban and surrounding non urban areas across India respectively, with daily air temperature at 850 hPa (T850), (**c**,**d**) same as (**a**,**b**) but for daily dewpoint temperature (DPT). Non-urban area around urban polygon was selected using 25 km buffer around urban polygon. The figure was developed using the Generic Mapping Tools (GMT, https://www.soest.hawaii.edu/gmt/).

Notwithstanding the limitation related to station based data availability for urban and non-urban areas to understand urban microclimate and its impact on rainfall extremes, we evaluated the scaling relationship for urban and surrounding non-urban areas using 3-hourly gridded rainfall from TRMM. We selected non-urban areas around urban polygon using 25 km buffer (from the urban center) and performed analysis on gridded data for urban and non-urban areas^64^. Since station based DPT is not available for non-urban areas, we used DPT from ERA-Interim reanalysis for estimating regression slopes between 3-hourly rainfall extremes in urban and surrounding non-urban areas. We found that regression slopes between T850/DPT and rainfall extremes in urban and non-urban areas are similar without any statistically significant (p > 0.05 using two-sided Rank Sum test) difference (Fig. 5). Moreover, scaling results for urban and non-urban regions obtained using QR and BT methods are consistent (Fig. S18). Our results are consistent with the findings of Mishra *et al*.^10^ who found no statistically significant (5% level) differences in mean regression slopes in urban and surrounding non-urban areas. While our results provide an initial assessment of the relationship between 3-hourly extremes and DPT/T850, long-term station data representing urban and non-urban regions will be valuable to understand the causes of higher scaling relationship in urban areas. Several factors including urban microclimate and urban heat island (UHI) can contribute to rainfall extremes in urban areas as shown in the previous studies^32, 73, 74^.

### Stationary and nonstationary return levels

Since daily and 3-hourly rainfall extremes show the super C-C relationship with DPT and T850, we used them (DPT and T850) as covariates of rainfall extremes under non-stationary conditions. We also estimate nonstationary design estimates using DPT and T850 covariates separately (Fig. S21). Both these covariates were found to have a correlation coefficient (r) less than 0.1 for all the urban areas for 1979–2015 indicating that they can be used together. Rainfall extremes in India showed mixed trends as reported in the previous studies^3, 64, 75–77^. However, it is important to note that nonstationarity conditions may not be evaluated merely on the basis of trends in time series. For instance, Yilmaz and Perera^78^ did not observe differences in stationary and nonstationary GEV models despite the presence of significant trends in extreme rainfall in Australia. We conducted the Priestley-Subba Rao (PSR) test to examine nonstationarity in rainfall time series for all 23 locations, which indicated non stationary nature of rainfall extremes at all the locations (Table S4).

We estimated differences in stationary and nonstationary design estimates of rainfall maxima for the 50 and100 year return period using daily annual maximum rainfall (AMR) from GSOD data for 1979–2015. We evaluated the differences in design estimates obtained from annual block maxima (ABM) approach and peak over threshold (POT) approach (taking top 35 independent rainfall events so that number of events in both the analysis are nearly the same) using the GSOD data (Fig. S19). We find that bias (%) in the design estimates obtained using POT and ABM approaches is within ±10%. Therefore, we used ABM approach for estimating stationary and nonstationary return levels. However, we acknowledge that a long-term record will be helpful to understand nonstationarity in a hydroclimatic time series^79^. Daily mean DPT and T850 were used as covariates to consider nonstationarity conditions in a nonstationary GEV model. Improvements of the nonstationary GEV model to estimate design values over a simple stationary GEV model was evaluated using the Chi-Square test at 5% significance level on negative log likelihood (nlh) estimates. We find that Deviance Statistic (D) calculated using nlh estimates obtained from stationary and nonstationary GEV models are greater than 3.84 [${\chi }_{1}^{2}(0.05)$ = 3.84] for all the locations, which provides a basis to use these covariates in the nonstationary GEV model (Table S2). Moreover, the goodness of fit of the non-stationary GEV model was tested for all the stations using probability and residual quantile plots (Fig. S20).

**Figure 6:**
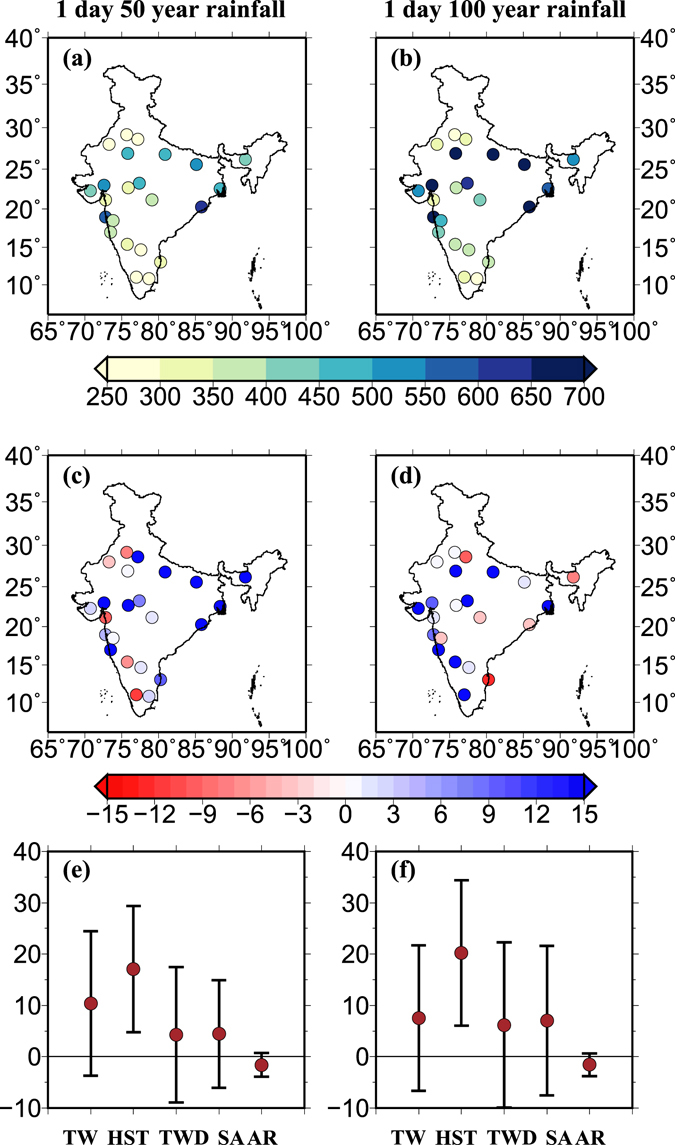
(**a**) 1 day 50 year rainfall maxima (in mm) for 23 cities across India assuming stationary conditions, (**b**) same as (**a**) but for 1 day 100 year rainfall maxima, (**c**) percentage bias in 1 day 50 year rainfall maxima considering stationary and nonstationary conditions, (**d**) same as (**c**) but for 1 day 100 year, (**e**) same as (**c**) but mean and standard deviation in percentage bias for different climatic zones and (**f**) same as (**d**) but for 1 day 100 year rainfall. DPT and T850 were used as covariate to account nonstationary conditions. Percentage change $(=\frac{NS-S}{NS}\times 100)$ in 1 day 50–100 year rainfall maxima were estimated using the stationary (S) and nonstationary (NS) conditions. Return values were estimated using ismev package in “R”. The figure was developed using the Generic Mapping Tools (GMT, https://www.soest.hawaii.edu/gmt/).

For 1 day 50 year rainfall maxima, 16 out of 23 locations showed increases (median 15.5%) due to the non-stationary conditions with covariates of DPT and T850 while 7 locations showed declines in rainfall maxima under the nonstationary conditions (Fig. 6a,b). Moreover, for 1 day 100 year rainfall maxima, 13 locations showed increases (median 18.3%) while 9 locations showed declines (median −3.5%) under the nonstationary conditions (Fig. 6c,d). Mean percentage change in 1 day 50 and100 year rainfall maxima was positive for the four out of five climatic zones under the nonstationary conditions (Fig. 6e,f). Change in 1 day 50 and100 year rainfall maxima was also estimated considering the stationary and nonstationary conditions using three sets of covariates: DPT and T850, DPT only, and T850 only. Using DPT and T850 together as covariates, percentage changes in rainfall maxima are higher as compared to using them separately (Fig. S21).

## Conclusions

Based on our findings the following conclusions can be made:

- The scaling relationship between rainfall extremes and SAT was negative in the majority of urban locations, which can be attributed to a negative relationship between air temperature and rainfall during the monsoon season. However, supper C-C scaling relationship between rainfall extremes and DPT/T850 was shown for the majority of urban locations in India that can be attributed to the convective nature of precipitation extremes over India. We find that SAT may not be sufficient to understand the changes in rainfall extremes over India in response to warming. Regression slopes obtained using the daily rainfall extremes against DPT (T850) were higher than the C-C rate for 20 (16) out of total 23 locations. Moreover, 3-hourly rainfall extremes from TRMM showed higher (the super CC relationship at 19 out of 23 locations) regression slopes with DPT and T850 indicating a higher sensitivity of sub-daily rainfall extremes.
- Regression slopes obtained using 3-hourly TRMM data against DPT and T850 were similar in urban and their surrounding non-urban areas. These results are based on observation stations that are located within 1–13 km of the city center and may not fully represent urban microclimate and other factors relevant to urban meteorology. However, long-term station based observations for urban and non-urban areas can provide robust estimates of the regression slopes and underlying causes for the sensitivity of rainfall extremes in urban and non-urban areas.
- Since daily and 3-hourly rainfall extremes showed a stronger relationship with DPT and T850, we considered them as covariates to evaluate the differences between stationary and non-stationary estimates of daily rainfall design storms intensities. We estimated differences in the stationary and nonstationary rainfall maxima for 50 and 100 year return periods using daily GSOD data considering DPT and T850 as covariates. We found rainfall maxima increased at a majority of locations under the nonstationary atmospheric conditions.

## Methods

### Data

We obtained daily rainfall data from the Global Summary of Day (GSOD) for the period of 1929–2015 for 100 stations in India mainly located in the vicinity of urban areas. The daily GSOD rainfall data is derived from the hourly observations contained in the Integrated Surface Hourly (ISH) dataset (DSI-3505) and is available from the National Oceanic and Atmospheric Administration (NOAA) website (ftp://ftp.ncdc.noaa.gov/pub/data/gsod/). Days that had less than 24 hours accumulated rainfall were removed. Moreover, we used stations with less than 10% missing data for any year during the period of 1979–2015. After the quality control, we finally selected 23 stations located in different climatic zones (Tropical Wet and dry, TWD; Humid Sub-Tropical, HST; Tropical Wet, TW; Semi-Arid, SA; Arid zone, AR) (Fig. 1a and Supplemental Table S1). Similarly, daily DPT data were obtained from the GSOD for 23 locations (for which rainfall data were available) in India for the period of 1979 to 2015.

We also obtained daily rainfall data at 0.05 degree resolution from Climate Hazards Group Infra-Red Precipitation with Station data (CHIRPS) for the grids that are closest to the urban areas for the period of 1981–2015^55, 56^. CHIRPS uses TRMM (TMPA 3B42 v7) to calibrate infrared Cold Cloud Duration (CCD) precipitation estimates which are further used in the ‘smart interpolation’ approach and blending procedure (using station based observations) to obtain long-term gridded global rainfall datasets^55, 56^. It is available for 50°S–50°N from 1981 to near-present and can be downloaded from ftp://ftp.chg.ucsb.edu/pub/org/chg/products/CHIRPS-2.0/. Katsanos *et al*.^80^ compared CHIRPS data with station data over Mediterranean basin and found a good correlation between them.

We obtained 3-hourly rainfall data at 0.25 degree resolution for the selected urban areas from the TRMM 3B42V7 (TRMM) for the period of 1998–2015^52^. TRMM is a gridded satellite product which uses passive microwave (PMW) data where it is available and infrared (IR) elsewhere since microwave radiance has a stronger relationship with precipitation than IR^52, 57^. TRMM is available from January 1998 onwards over 50°S–50°N and 180°W–180°E and can be downloaded from http://disc. sci.gsfc.nasa.gov/SSW/. We used TRMM 3B42V7 as sub-daily station data are unavailable and it is more reliable than other available multi-satellite rainfall products over India^54^. Moreover, TRMM 3B42V7 (TRMM onwards) captures spatial and temporal features of rainfall well against rain gauge measurements as shown by Shah and Mishra^81^.

We used daily air temperature data at 850 hPa (T850) obtained from the latest global atmospheric ERA-Interim reanalysis data which is produced by the European Centre for Medium-Range Weather Forecasts (ECMWF) with the Integrated Forecast System at a T255 spectral resolution on 60 vertical levels which reaches from the surface up to 0.1 hPa^82^. The data is available from January 1979 to present and can be downloaded from http://apps.ecmwf.int/datasets/data/interim-full-daily/levtype=pl/. The data was regridded at 0.25 degree resolution using bilinear interpolation. We used T850 (roughly 1.5 km) sufficiently above the boundary layer of the atmosphere instead of SAT in order to avoid the effects of ground geographical features (like the sea) on air temperature. Moreover, SAT data are strongly correlated with the monsoon season rainfall, which might introduce bias in the scaling process.

Since reanalysis products have uncertainties, we used air temperature at 850 hPa from the two other reanalysis products. The first one is obtained from the National Centres for Environmental Prediction (NCEP) Climate Forecast System Reanalysis (CFSR). CFSR is a high resolution (0.5 degree) global dataset which is developed using a coupled atmospheric-ocean-land-surface system^83^. It is available at 64 levels extending from surface to 0.26 hPa. CFSR is available from 1979 onwards and can be downloaded from http://rda.ucar.edu/datasets/ds093.1/. The second dataset for T850 was obtained from the Modern-Era Retrospective analysis for Research and Applications version 2 (MERRA2). MERRA2 is global hourly data at 0.5 × 0.625 degree resolution, available from 1980 onwards and can be downloaded from https://gmao.gsfc.nasa.gov/reanalysis/MERRA-2/. The MERRA2 has improvements over MERRA datasets because it assimilates modern hyperspectral radiance and microwave observations along with GPS- Radio Occultation datasets^84^. The hourly values in a day were averaged to find mean daily temperature at 850 hPa.

### Analysis

We extracted datasets (rainfall and DPT) for 23 urban areas from the daily GSOD data. However, gridded datasets for the urban locations were selected in such a way so that centre of grid lies within a urban area. Moreover, datasets were at different spatial resolution, therefore, we applied areal reduction factors (ARF) based on U.S. Weather Bureau 1975 method^85, 86^ (TP-29) to bring them to point scale, which is given by

1 $$AR{F}_{TP-29}=\frac{\frac{1}{n}\sum _{j=1}^{n}{\hat{R}}_{j}}{\frac{1}{k}\sum _{i=1}^{k}(\frac{1}{n}\sum _{j=1}^{n}{R}_{ij})}$$

where ${\hat{R}}_{j}$ is the annual maximum areal rainfall for year *j*, *R*_*ij*_ is the annual maximum point rainfall for year *j* at station *i*, *k* is the number of stations in the area, and *n* is the number of years^85, 86^. The choice of this ARF from different ARFs is discussed in Supplemental Information.

Regression slopes (scaling) were estimated by both binning technique (BT) and quantile regression (QR). In binning technique (BT), rainfall data is matched to temperature data for each day and is classified into bins of increasing temperature either of equal temperature bin size^10, 15, 21, 33, 45^. However, bin size and outlying data in each bin may affect the scaling estimates^27^. Therefore, we used quantile regression which is more robust and flexible method and does not require such assumptions^28, 43, 44^.

In quantile regression (QR), for a set of data pairs (*x*_*i*_, *y*_*i*_) for *i* = 1, 2, …., *n*, the quantile regression for a given percentile *p*(95^th^ percentile in our study) is expressed as

2 $${y}_{i}={\beta }_{0}^{(p)}+{\beta }_{1}^{(p)}{x}_{i}$$

where *y*_*i*_ is logarithmically transformed rainfall^45^, *x*_*i*_ corresponding temperature (SAT/T850/DPT) and regression slope of rainfall with temperature in percentage (dR95/K, %) is estimated using exponential transformation of regression coefficient ${\beta }_{1}^{(p)}$ ^28^:

3 $$dR95( \% )/K=100.({e}^{{\beta }_{1}^{(p)}}-1)$$

More information on this method can be obtained from Koenker and Basset^43^. We carried out Quantile regression analysis using ‘quantreg’ package in statistical programming language ‘R’^87, 88^. Since this package does not give R^2^ value to estimate goodness of fit, we used pseudo R^2^ value described by Koenker and Machado^89^.

To check the robustness of our results, we also estimated regression slope using a binning technique (BT). We, therefore, used the method of Mishra *et al*.^10^ to establish the scaling relationship of extreme rainfall with SAT, DPT, and T850. The same method has been used in many previous studies^20, 22, 33, 68^. For each station, we extracted wet events (rainfall > = 1 mm) for all days in a year and their corresponding daily mean DPT from GSOD dataset. The data were then placed into 20 temperature bins (based on daily mean DPT) of approximately same size, sorted from the lowest to highest temperature values. Further, for each temperature bin, we estimated the 95^th^ percentile of rainfall (R95) and daily mean dewpoint temperature (DPT). Then, we fitted a linear regression on the logarithm of R95 and DPT. The percentage change in R95 (dR95%/K) with respect to change in DPT (regression slopes onwards) was estimated using regression equation between the lowest (mean dewpoint temperature of the first bin) and highest dewpoint temperature (DPT_R95_, peak point temperature) where R95 maxima occurred. Similarly, we scaled extreme rainfall from the other datasets (GSOD, TRMM, and GPM) available at daily, 3-hourly, and half-hourly resolutions with daily mean SAT, DPT and T850 respectively.

We obtained polygons of selected urban areas using urban extent map of global cities which are based on MODIS 1 km land cover data^90^ and a non-urban area around urban polygon was selected using 25 km buffer^64^. 3-hourly TRMM data was extracted for urban and non-urban areas so that their grid centres lie within respective polygons and scaling was done against T850 and DPT.

In order to examine nonstationarity of rainfall time series we used the Priestley-Subba Rao (PSR) test^91^ using the “fractal” package in the statistical programming language ‘R’. This test is based on an evolutionary spectral analysis which examines the homogeneity of evolutionary spectra with time, and a p-value for T decides stationarity/nonstationarity of a time series. We used the Generalised Extreme Value (GEV) distribution to estimate stationary and nonstationary return levels (design values) of extreme rainfall events in the urban areas. The GEV distribution has three parameters: location parameter (*µ*), scale parameter (*σ*) and shape parameter (*k*) for the extremes time series^10, 64^. We used a block maxima approach (ABM; annual maximum rainfall time series and their corresponding dew point temperature and air temperature) to fit the GEV distribution using maximum likelihood estimates (MLE) with the help of gev.fit function from “ismev” package in ‘R’^66, 92, 93^. We also used a peak over threshold (POT) approach to obtain design estimates since there are chances of missing rainfall extremes using ABM approach. For this, we used the top 35 rain events during the entire period (and their corresponding dew point temperature and air temperature) and used generalized pareto distribution^94^ (GPD) to estimate design estimates using the gpd.fit function in MATLAB.

Under the stationary assumption no covariate was considered, hence, all the three distribution parameters were constant for the entire period of analysis. Under the nonstationary assumption, DPT and T850 were taken as covariates and location parameter (*µ*) was allowed to vary linearly with the both covariates and remaining two parameters (*σ* and *k*) kept constant^92, 95^. We evaluated improvements in the nonstationary model over the simple stationary model using Deviance Statistic (*D* = 2{*l*_1_(*M*_1_) − *l*_*o*_(*M*_*o*_)}), which was calculated using maximised log-likelihood under models considering stationary (*l*_1_(*M*_1_)) and nonstationary (*l*_*o*_(*M*_*o*_)) assumptions. If *D* > 3.84 (i.e. chi-square test at 5% significance level) then nonstationary model can be accepted, which also justifies the use of covariates in nonstationary model^88^. More details on this method can be obtained from Katz *et al*.^96^ and at http://www.ral.ucar.edu/~ericg/softextreme.php.

## Electronic supplementary material

Contrasting response of rainfall extremes to increase in surface air and dewpoint temperatures at urban locations in India
