# Supplementary material for: Contrasting response of rainfall extremes to increase in surface air and dewpoint temperatures at urban locations in India
Source: Sci Rep. 2017 Apr 27;7:1228. doi: 10.1038/s41598-017-01306-1 (PMC5430704; doi:10.1038/s41598-017-01306-1)
Supplement: Supplementary file 1 — Contrasting response of rainfall extremes to increase in surface air and dewpoint temperatures at urban locations in India [file 41598_2017_1306_MOESM1_ESM.pdf]

Supplemental Information

Contrasting response of rainfall extremes to increase in surface air and dewpoint temperatures at urban locations in India

Haider Ali and Vimal Mishra*

Civil Engineering

Indian Institute of Technology (IIT) Gandhinagar, Gujarat, India

Corresponding author: vmishra@iitgn.ac.in

Areal Reduction Factors

We used areal reduction factors (ARF) to convert gridded rainfall (TRMM, CHIRPS) to point estimates. ARF is defined as the ratio of the average areal depth of rainfall and the average point depth, which can range from 0 to 1[1,2]. We considered five different ARFs in our study as listed below:

a) ***U.S. Weather Bureau 1957 method (TP-29; arf1)***: In this method, ARF values are estimated as a ratio of an average annual-maximum areal precipitation depth for a given period to the average annual-maximum point precipitation depth in the area for the same period[2-4]. This method assumes that the relationship between depth and area is not influenced by the recurrence interval (frequency) of the point rainfall[1].

$$ARF_{TP-29} = \frac{\frac{1}{n}\sum_{j=1}^{n}\hat{R}_j}{\frac{1}{k}\sum_{i=1}^{k}\left(\frac{1}{n}\sum_{j=1}^{n}R_{ij}\right)} \qquad (1)$$

where $\hat{R}_j$ is the annual maximum areal rainfall for year *j*, R_{ij} is the annual maximum point rainfall for year *j* at station *i*, *k* is the number of stations in the area, and *n* is the number of years[2].

b) ***Storm-centred ARFs (arf2)***: In this method, ARF is defined as a ratio of maximum areal rainfall (P_{area}) within the storm zone for a given area and duration to maximum point rainfall (P_{point}) within the same storm for the same duration period[2-4].

$$ARF = \frac{P_{area}}{P_{point}} \qquad (2)$$

c) ***Leclerc and Schaake method (1972; arf3):*** ARF is given by

$$ARF = \frac{Z_E}{Z_T} = 1 - e^{-1.1t^{0.25}} + e^{(-1.1t^{0.25} - 0.01A)} \qquad (3)$$

where Z_E is areal average effective precipitation, Z_T is total point precipitation, A is area in square kilometer and *t* is temporal resolution of observed data[5,6].

d) ***ARF used in United Kingdom (NERC, 1975; arf4)***: ARF is given by

$$ARF_{FSR} = \frac{1}{IJ}\sum_j \sum_i \frac{P'_{ij}}{P_{ij}} \qquad (4)$$

where P'_{ij} is the annual maximum areal rainfall over a station i and year j, P_{ij} is the annual maximum point rainfall at each station i for year j, A is the area of region, I is the total number of stations in the region, and J is the record length (in years)[4].

e) ARF based on regression method (arf5): This arf is derived with a power best-fit regression method and is expressed by

$$ARF = 1.09(a^{-0.0667}) \qquad (5)$$

where a is the area in square kilometer[7].

We used gridded data from CHIRPS/Daily TRMM and applied these five different ARFs to estimate 90 to 99.9 th percentiles of rainfall. We then compared these percentile values from those obtained using GSOD and estimated root mean square error (RMSE) for each location (Supplemental Table S2). We find that for *arf1* RMSE is minimum and it is maximum for *arf3*. Moreover, we estimated regression slopes using rainfall from CHIRPS and Daily TRMM with DPT/T850 for i) without applying any ARF, ii) with the best ARF method (*arf1*), and iii) with the worst ARF method (*arf3*) (Supplemental Figure S1). We observed similar scaling results for these three cases for the majority of locations, which shows that scaling results are weakly dependent on the choice of ARF for the selected locations. However, we still used arf1 for converting gridded rainfall (from TRMM and CHIRPS) to point scale in our study.

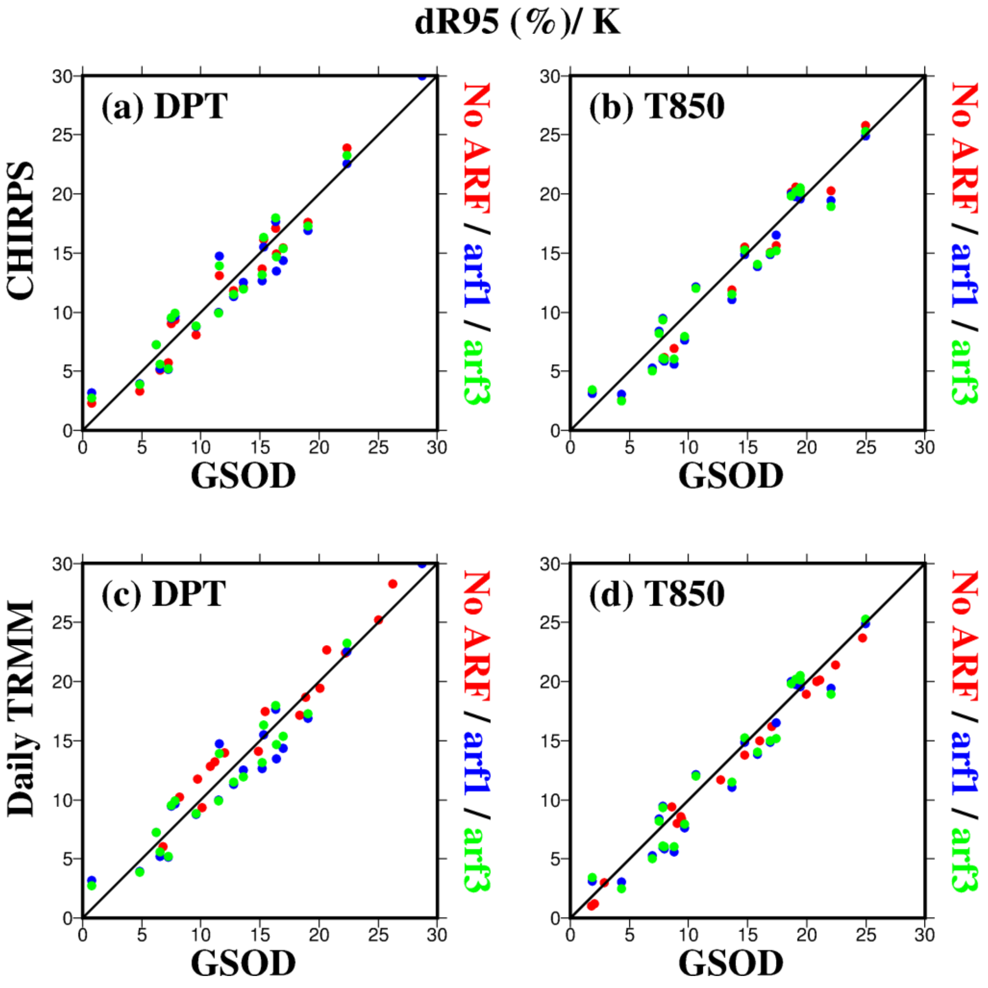

Supplemental Figure S1 (a) Agreement in scaling (dR95/K, %) results between GSOD and CHIRPS with DPT, pooled for all 23 urban areas after applying no ARF (red), *arf1* (best ARF; blue) and *arf3* (worst ARF; green) on gridded CHIRPS data, (b) same as (a) but for T850 and (c-d) same as (a-b) but for Daily TRMM data. The figure was developed using the Generic Mapping Tools (GMT, https://www.soest.hawaii.edu/gmt/).

Mean sea level pressure (SLP) and T850 composites

We obtained mean sea level pressure (SLP) data from the ERA-Interim reanalysis data for the period of 1979-2015. To understand the sea level pressure variability during the extreme rainfall events in urban areas, we developed composite maps of SLP and T850 anomalies. Similar to the method of Mishra et al.[8], top 100 extreme rainfall events during the period of 1979-2015 for each station were selected and SLP and T850 for each day of extreme rainfall events were extracted. T850 anomalies were estimated using the mean of the corresponding day for 30 years. Using T850 anomalies and SLP, we constructed composites for each city and different climatic zones taking mean of data for all the urban areas within that region. We also obtained moisture convergence data from the ERA-Interim reanalysis and developed composites of moisture convergence anomalies for the only top event for the period 1979-2015 for one representative urban area in each climatic zone. We did not consider more than 1 event for moisture convergence composites as we found that maps with more events showed a little spatial variability probably due to different directions of the moisture transport. However, analysis of the top most extreme rainfall event showed the presence of moisture anomaly during the heavy rainfall event.

Supplemental Figure S2a shows daily mean T850 anomalies of 3K for the TWD climatic zone with positive anomalies in northwest and negative anomalies in the northeast and southern India. This explains precipitation growth associated with the ascent of air which developed in the frictional layer between cold and warm front[8]. The temperature gradient which is formed due to the movement of cold front results in rainfall. Kunkel et al.[9] reported that extreme rainfall increases with increase in temperature contrast between the two (cold and warm) air masses. Moreover, mean SLP pattern is low centred with a trough extending

towards southern India which resembles a cold front as shown in Mishra et al.[8]. Similar patterns were observed for the HST, TW, AR and SA climatic zones (Fig. S2b, d and e respectively). However, for the TW climatic zone, positive anomalies were spread over the most parts of India with negative anomalies in the northeast and southwest (Fig. S2c). We do not find any substantial variation in the standard deviation of T850 anomalies for the top 100 rainfall events in urban areas within three climatic zones: TW, HST and TWD (Fig. S2f). The composite maps were developed for each urban areas to analyse the SLP and T850 conditions during extreme rainfall events (Fig. S3). We also developed moisture convergence anomalies composites using the convergence data from the ERA-Interim reanalysis for the top rainfall events for one city in each climatic zone to show the role of moisture availability in the occurrence of rainfall extremes (Fig. S4). We notice positive moisture convergence anomalies in the region where urban areas are located and negative anomalies in regions away from urban areas indicating the moisture transport during the extreme rainfall events.

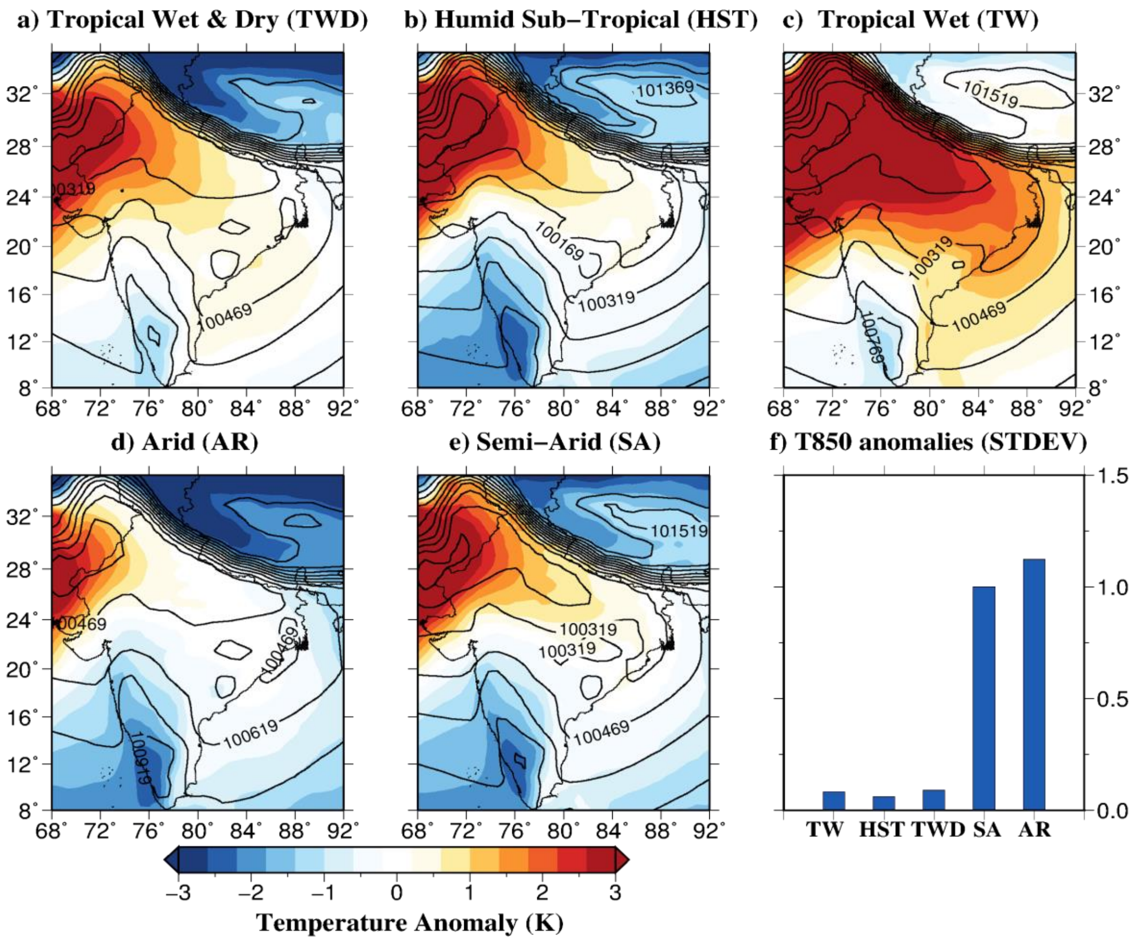

Supplemental Figure S2 (a-e) SLP and mean T850 anomalies composites for top 100 extreme rainfall events for the period of 1979-2015 for different climatic zones, (f) standard deviation in mean T850 anomalies for top 100 rainfall events for cities within different climatic zone. The figure was developed using the Generic Mapping Tools (GMT, https://www.soest.hawaii.edu/gmt/).

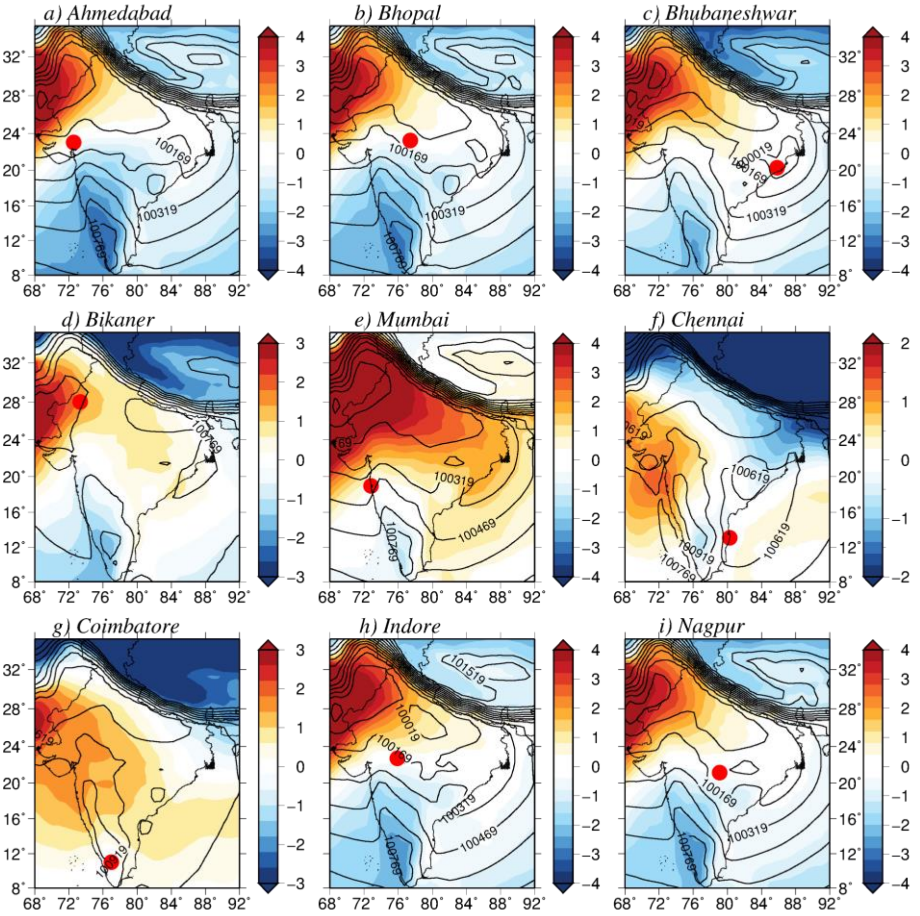
a) Ahmedabad
b) Bhopal
c) Bhubaneshwar
d) Bikaner
e) Mumbai
f) Chennai
g) Coimbatore
h) Indore
i) Nagpur

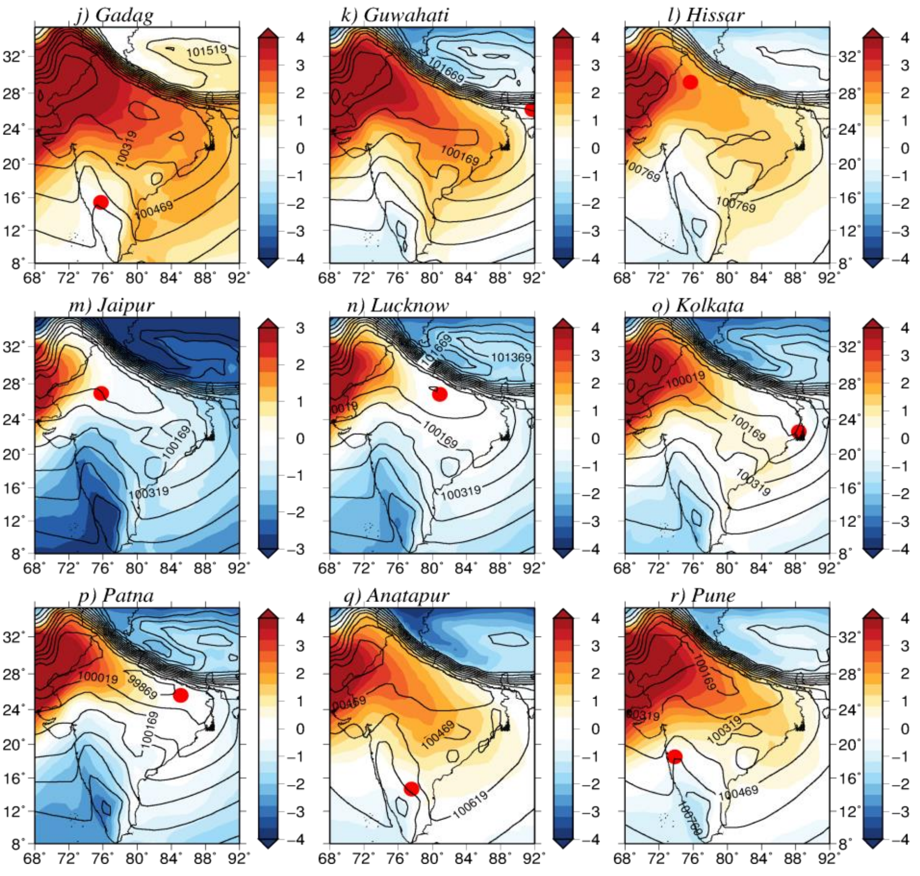
j) Gadag
k) Guwahati
l) Hissar
m) Jaipur
n) Lucknow
o) Kolkata
p) Patna
q) Anatapur
r) Pune

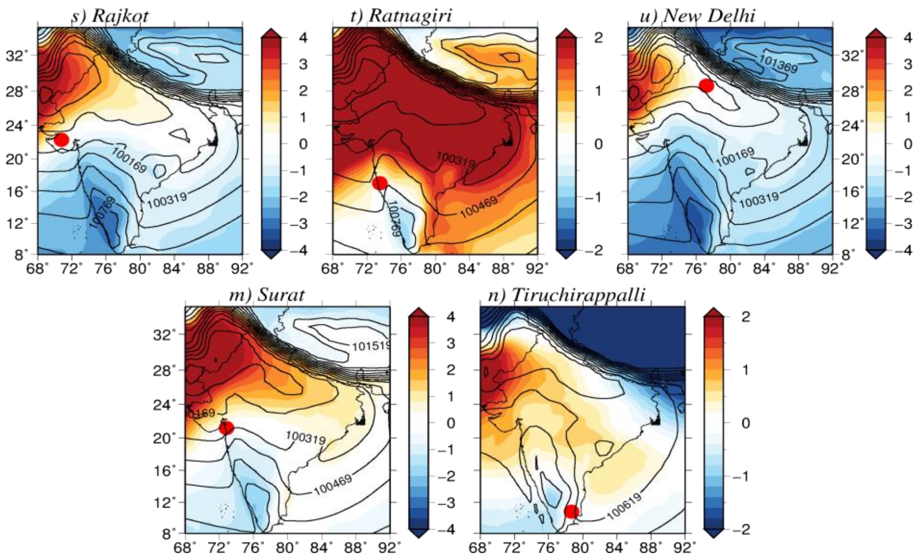

Supplemental Figure S3. Same as supplemental Figure S2 but for individual urban areas. The figure was developed using the Generic Mapping Tools (GMT, https://www.soest.hawaii.edu/gmt/).

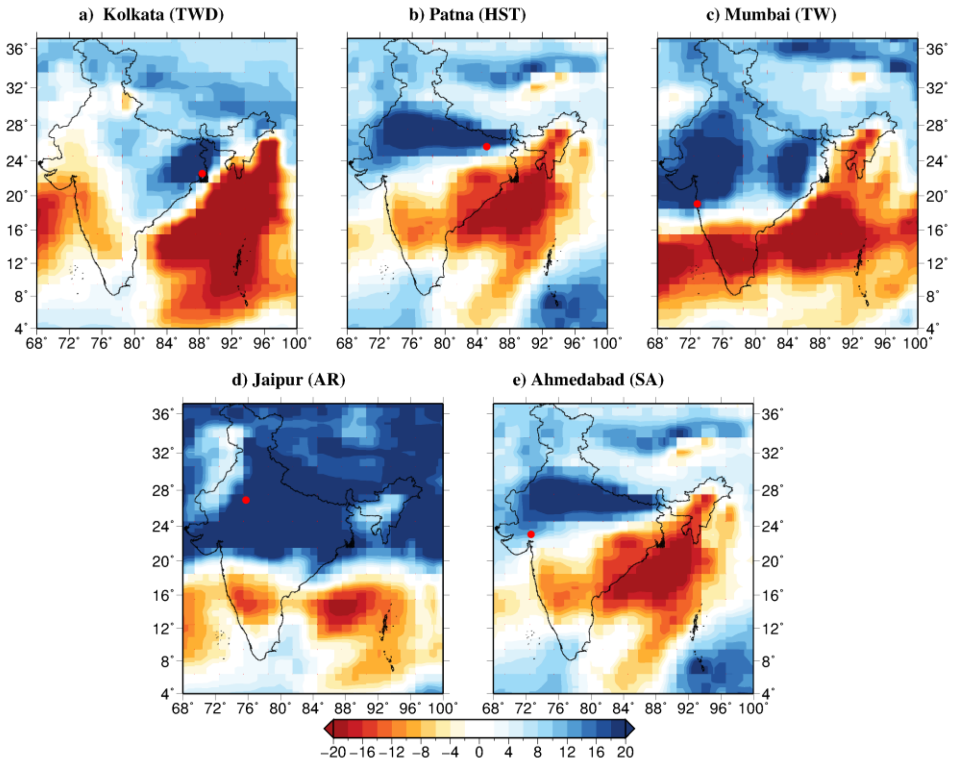

Supplemental Figure S4 (a-e) Moisture convergence anomalies (mm/day) for the top extreme rainfall event for the period of 1979-2015 for one representative urban area in each climatic zones. The figure was developed using the Generic Mapping Tools (GMT, https://www.soest.hawaii.edu/gmt/).

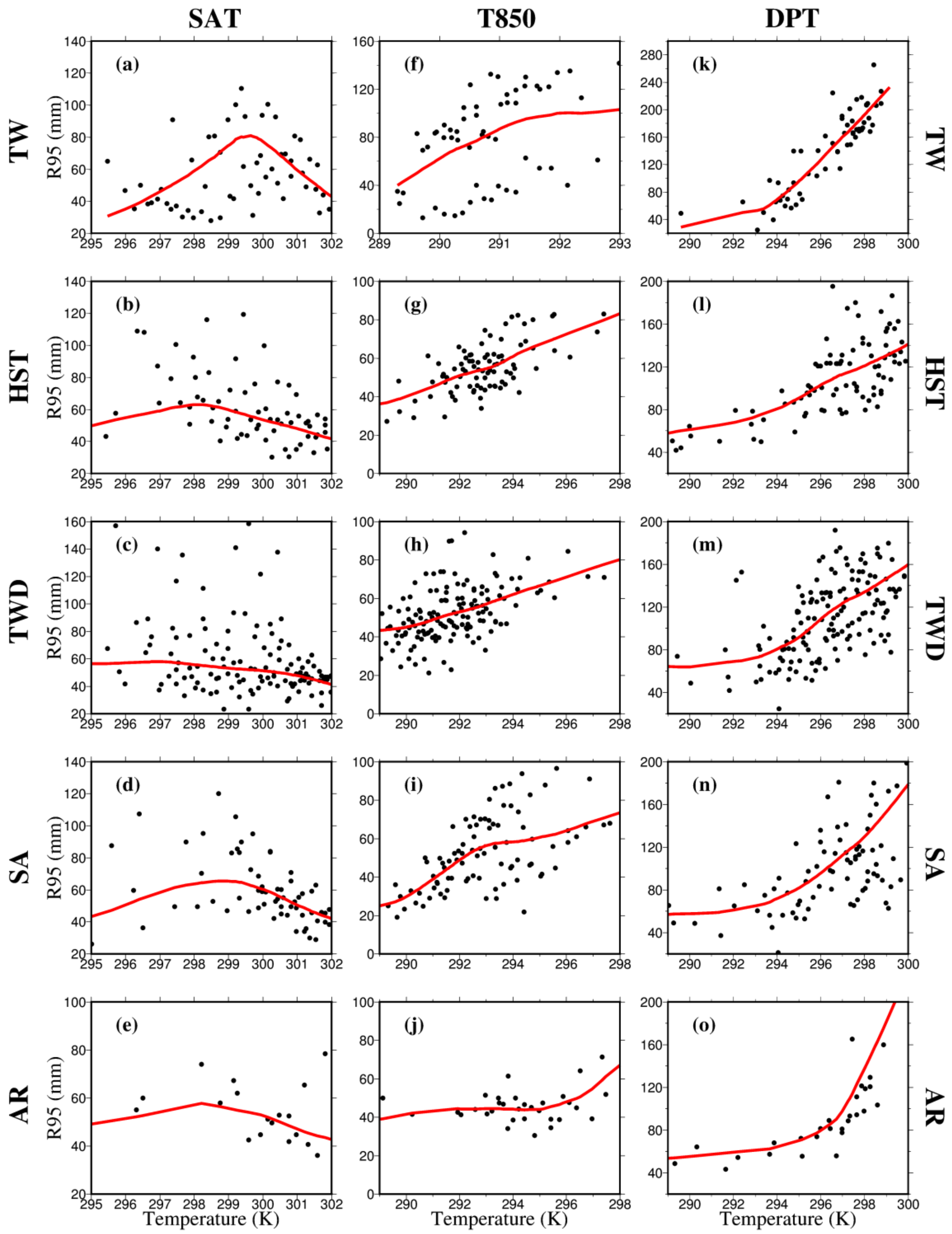

Supplemental Figure S5(a-e) Relationship between rainfall extremes (R95) obtained from daily GSOD with daily surface air temperature (SAT) for all the climatic zones: TW, HST, TWD, SA and AR respectively for the period of 1979-2015, (f-j) same as (a-e) but for daily air temperature at 850 hPa (T850), and (k-o) same as (f-j) but for daily dewpoint temperature (DPT). Red lines indicate fitted lines estimated using LOWESS. The figure was developed using the Generic Mapping Tools (GMT, https://www.soest.hawaii.edu/gmt/).

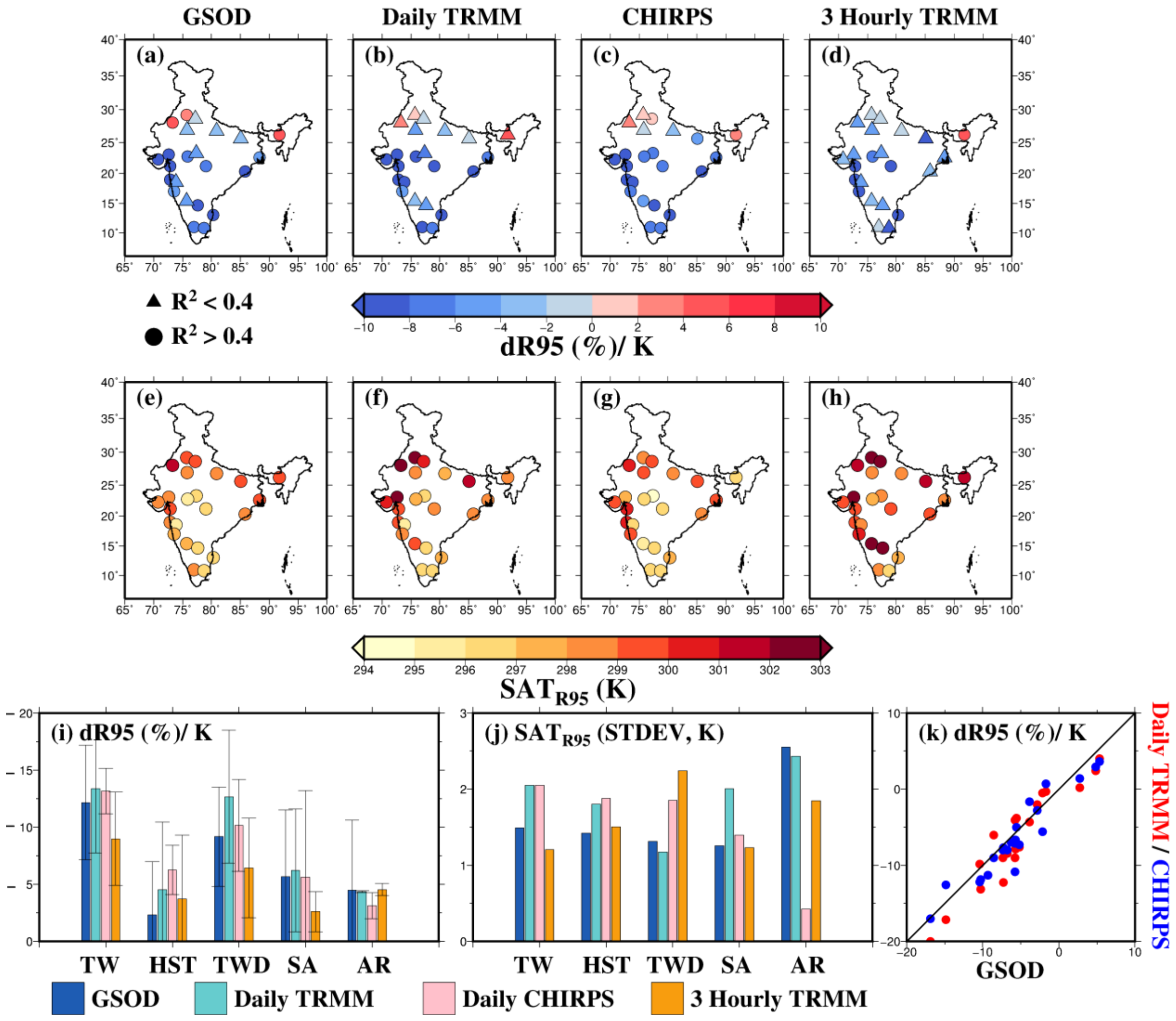

Supplemental Figure S6 (a-d) Regression slopes (dR95/K, %) of extreme rainfall obtained from daily GSOD, daily TRMM, daily CHIRPS and 3-hourly TRMM data, respectively with surface air temperature (SAT) for 23 urban areas across India using binning technique (BT), (e-h) peak point temperature (SAT_{R95}) for selected urban areas for same datasets respectively, (i) regression slopes (dR95/K) from daily GSOD (blue), daily TRMM (cyan), daily CHIRPS (pink) and 3-hourly TRMM (orange) data for different climatic zones respectively where bars denote mean values and whiskers show standard deviations, (j) same as (i) but for standard deviations (STDEV) in SAT_{R95}, (k) agreement in scaling results between GSOD and Daily TRMM (red) and GSOD and CHIRPS (blue), pooled for all 23 urban areas. The figure was developed using the Generic Mapping Tools (GMT, https://www.soest.hawaii.edu/gmt/).

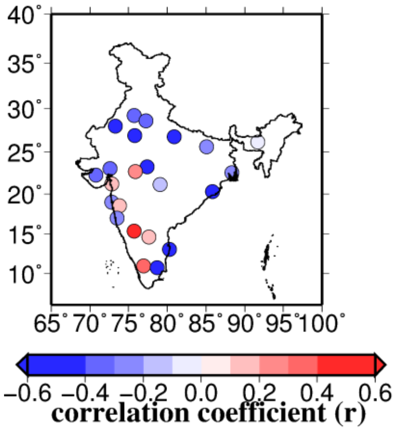

Supplemental Figure S7. Correlation coefficient (r) of total rainfall obtained from daily GSOD with mean surface air temperature (SAT) for monsoon season (June-September) for the period of 1979-2015 for 23 urban areas across India. The figure was developed using the Generic Mapping Tools (GMT, https://www.soest.hawaii.edu/gmt.

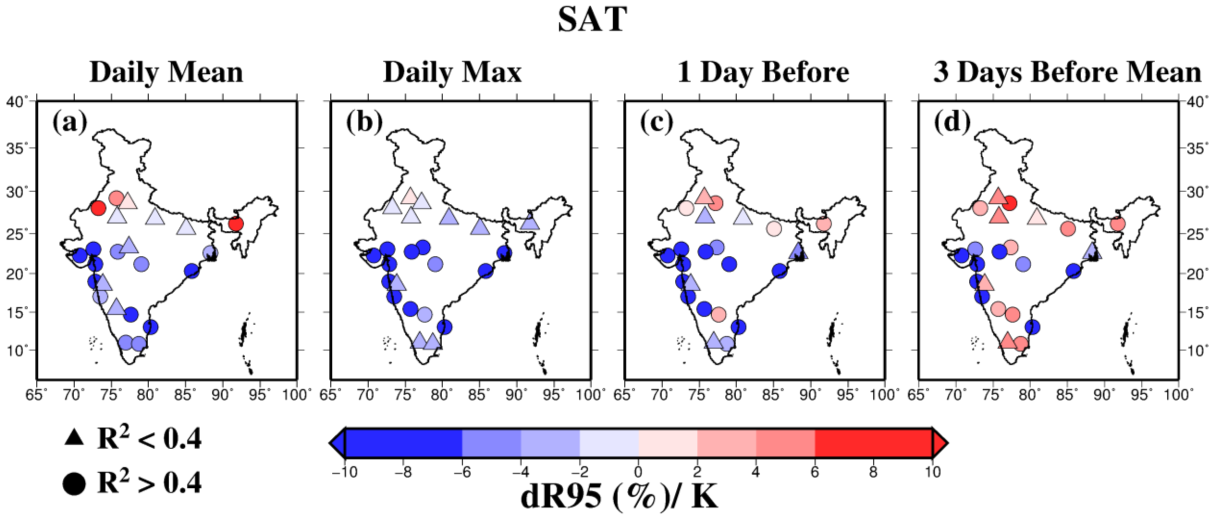

Supplemental Figure S8. Regression slopes (dR95/K, %) of extreme rainfall obtained from daily GSOD data with (a) daily mean SAT, (b) daily maximum SAT, (c) daily mean SAT for 1 day prior to rain event and (d) daily mean SAT for 3 days prior to rain event. The figure was developed using the Generic Mapping Tools (GMT, https://www.soest.hawaii.edu/gmt/).

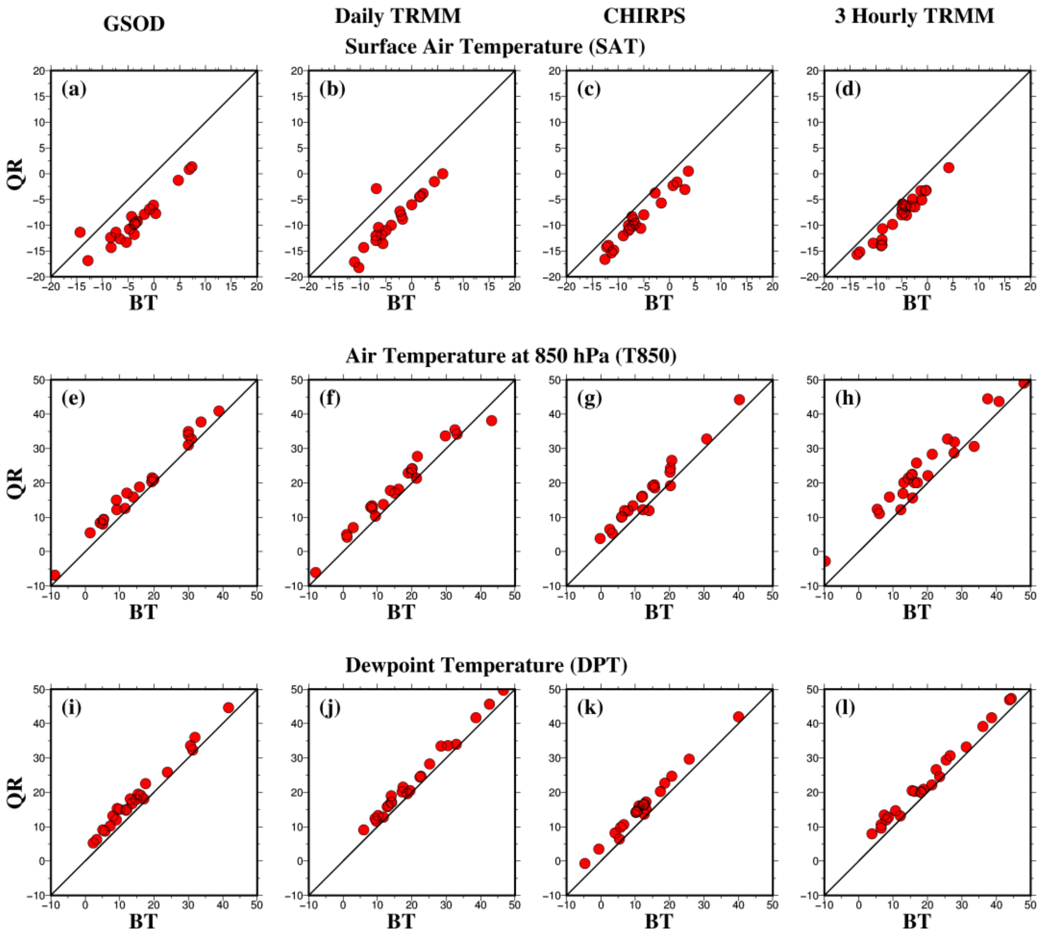

Supplemental Figure S9. (a-d) Agreement in scaling (dR95/K, %) results between binning technique (BT) and quantile regression (QR) for GSOD, Daily TRMM, CHIRPS and 3-hourly TRMM respectively with daily surface air temperature (SAT), pooled for all 23 urban areas, (e-f) same as (a-d) but for daily air temperature at 850 hPa (T850), and (i-l) same as (e-f) but for daily dewpoint temperature (DPT). The figure was developed using the Generic Mapping Tools (GMT, https://www.soest.hawaii.edu/gmt/).

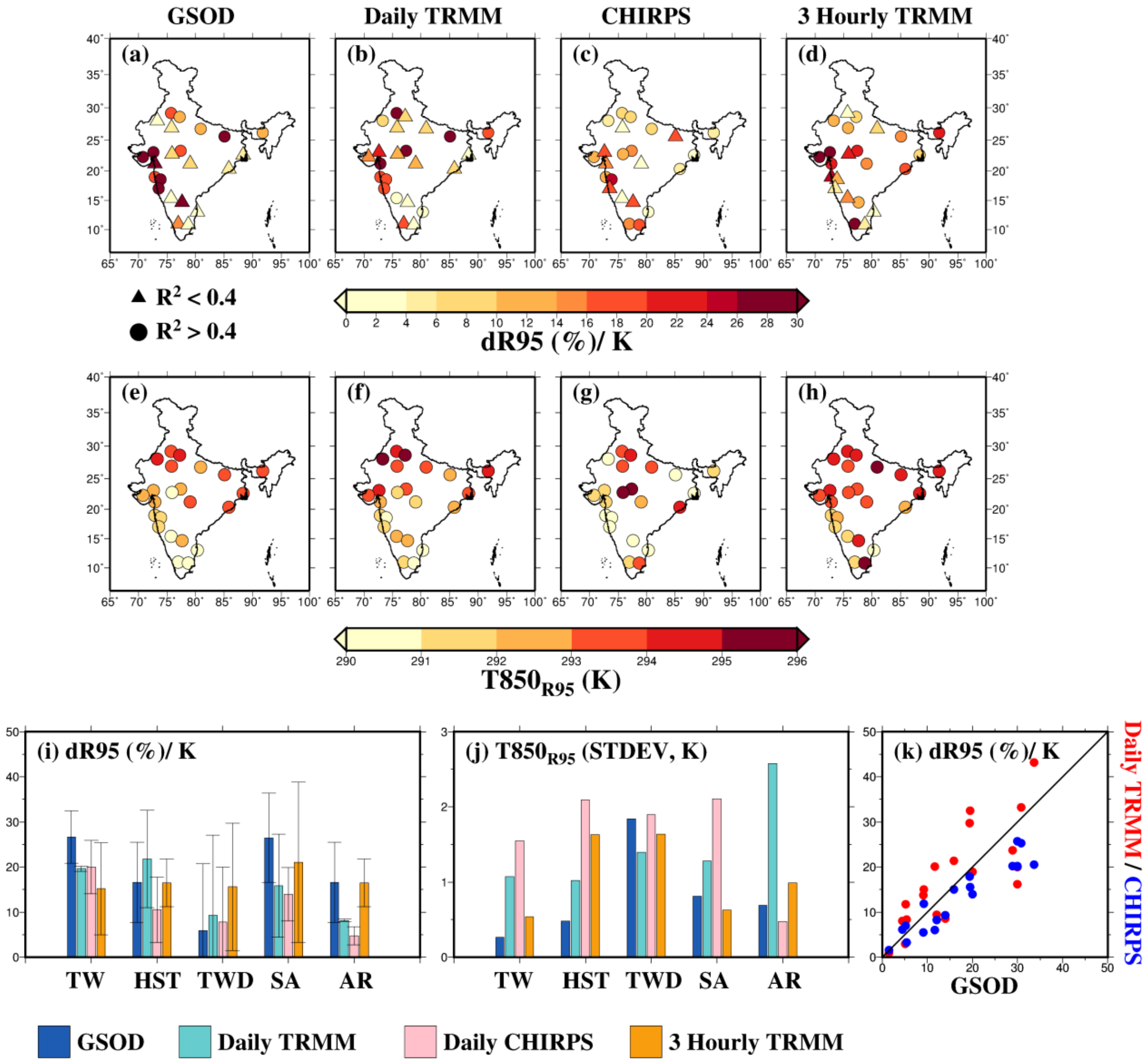

Supplemental Figure S10 (a-d) Regression slopes (dR95/K, %) of extreme rainfall obtained from daily GSOD, daily TRMM, daily CHIRPS and 3-hourly TRMM data, respectively with air temperature at 850 hPa (T850) for 23 urban areas across India using binning technique (BT), (e-h) peak point temperature ($T850_{R95}$) for selected urban areas for same datasets respectively, (i) regression slopes (dR95/K) from daily GSOD (blue), daily TRMM (cyan), daily CHIRPS (pink) and 3-hourly TRMM (orange) data for different climatic zones respectively where bars denote mean values and whiskers show standard deviations, (j) same as (i) but for standard deviations (STDEV) in $T850_{R95}$, (k) agreement in scaling results between GSOD and Daily TRMM (red) and GSOD and CHIRPS (blue), pooled for all 23 urban areas. The figure was developed using the Generic Mapping Tools (GMT, https://www.soest.hawaii.edu/gmt/).

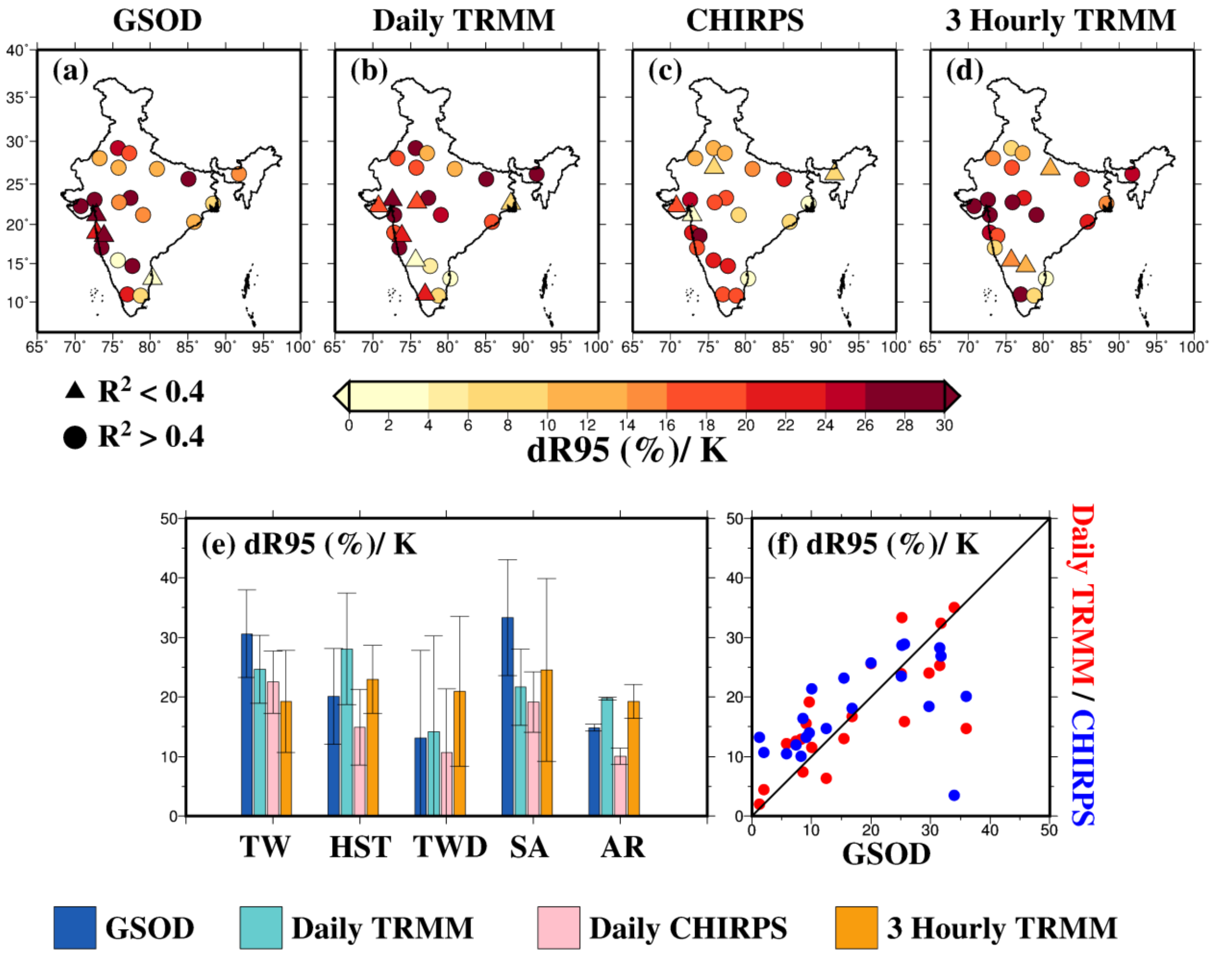

Supplemental Figure S11. Same as Figure2 but for T850 obtained from MERRA2 reanalysis data using quantile regression (QR). The figure was developed using the Generic Mapping Tools (GMT, https://www.soest.hawaii.edu/gmt/).

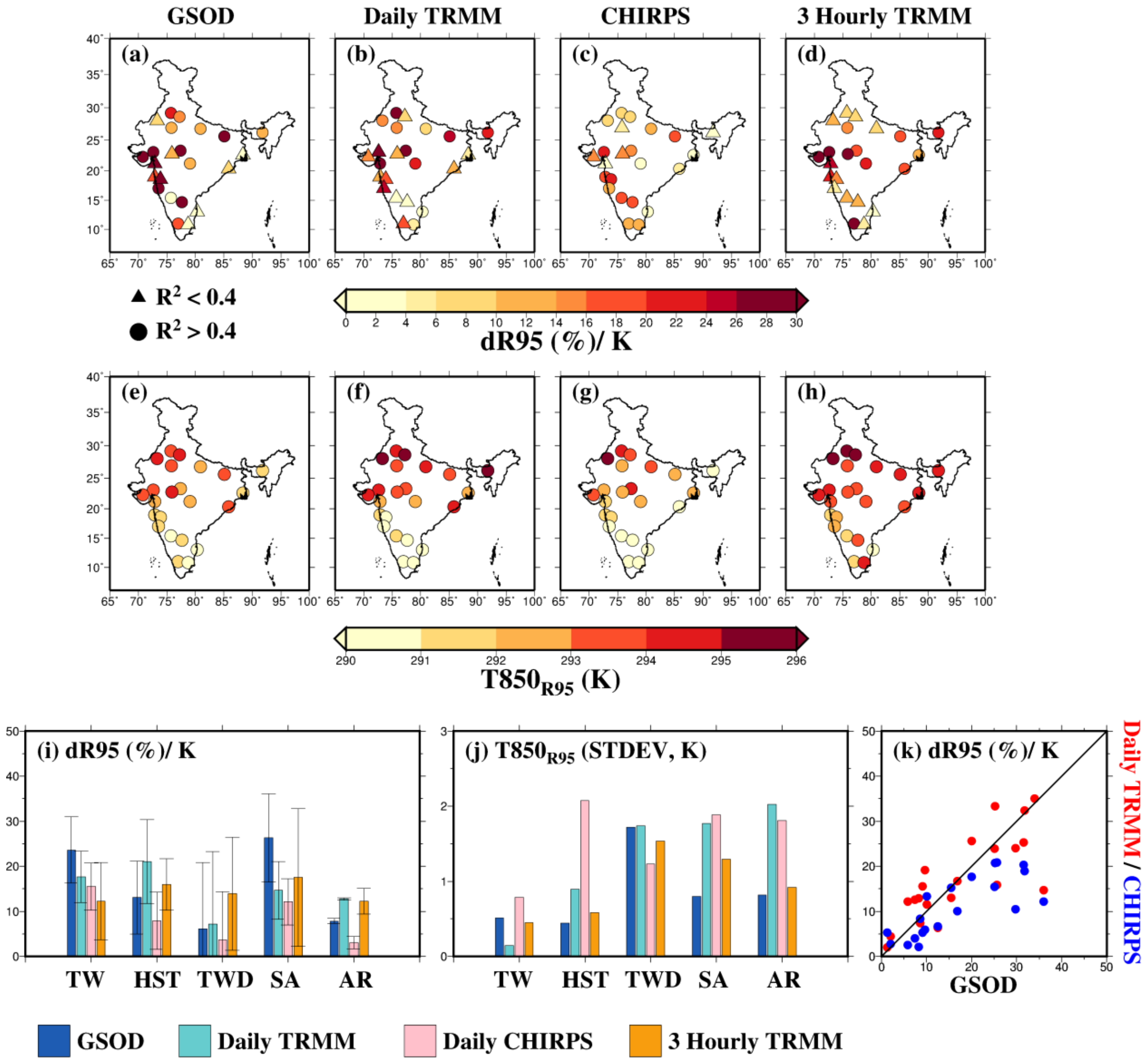

Supplemental Figure S12. Same as Supplemental Figure S11 but for T850 obtained from MERRA2 reanalysis data using binning technique (BT). The figure was developed using the Generic Mapping Tools (GMT, https://www.soest.hawaii.edu/gmt/).

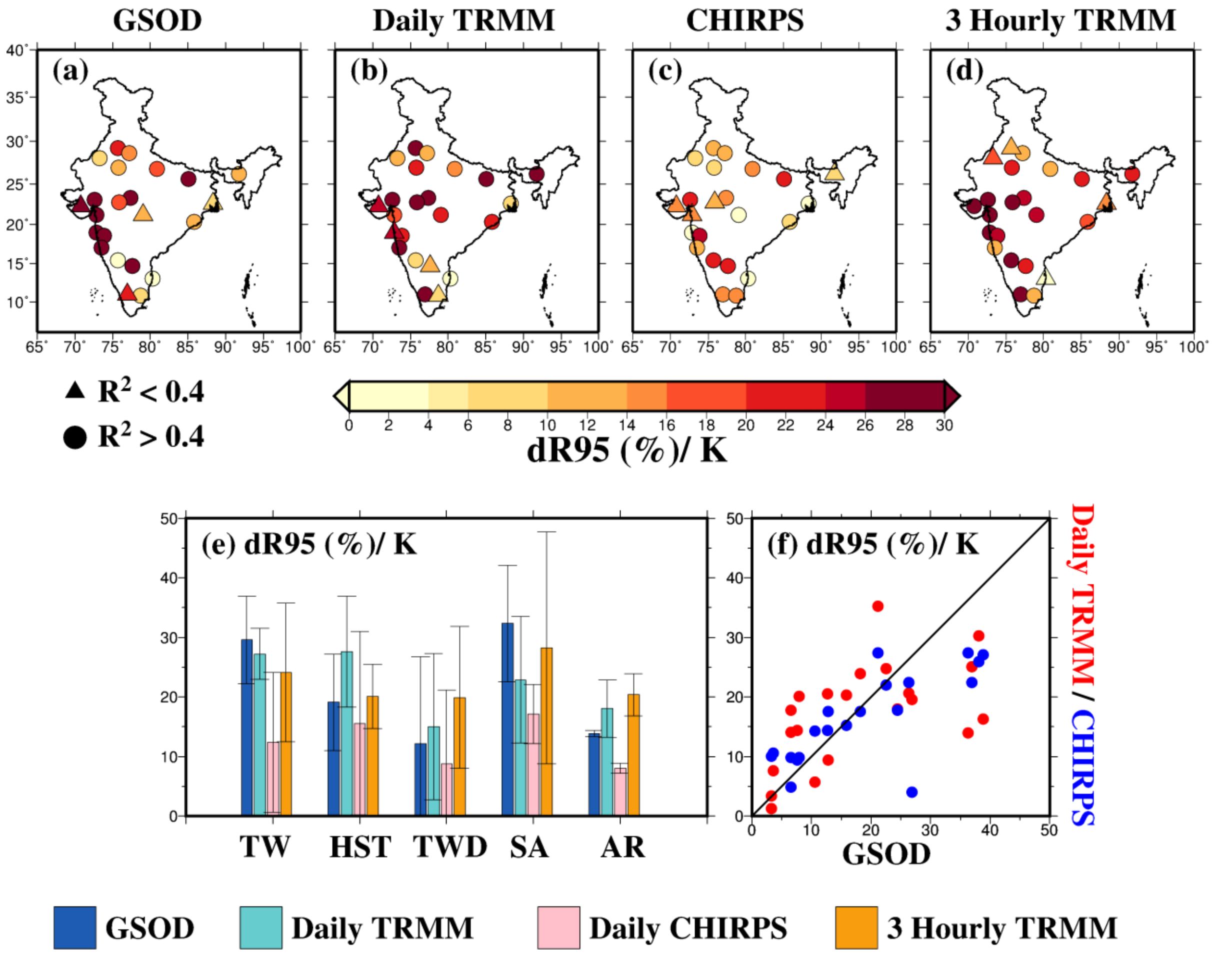

Supplemental Figure S13. Same as Figure2 but for T850 obtained from CFSR reanalysis data using quantile regression (QR) method. The figure was developed using the Generic Mapping Tools (GMT, https://www.soest.hawaii.edu/gmt/).

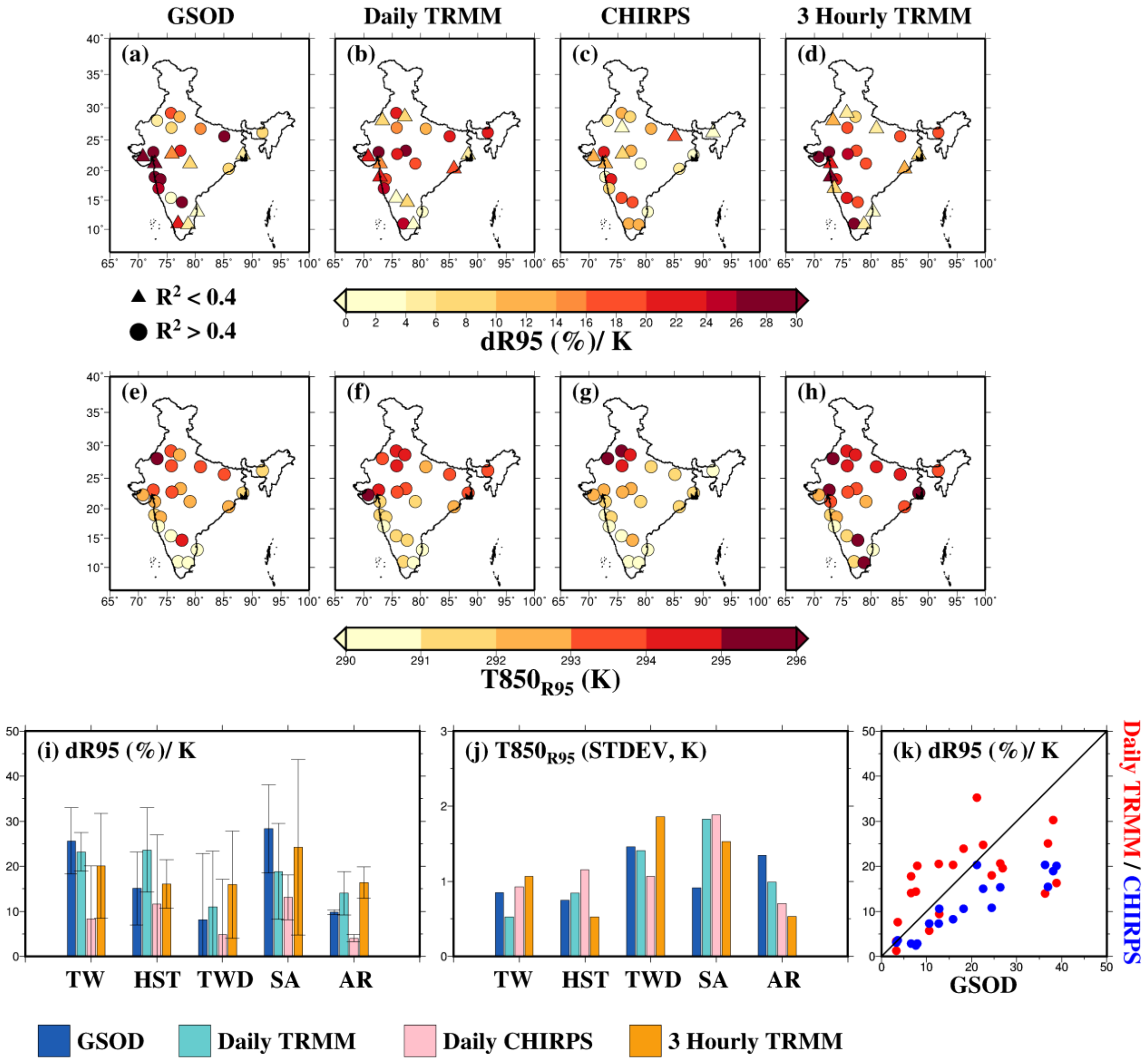

Supplemental Figure S14. Same as Supplemental Figure S11 but for T850 obtained from CFSR reanalysis data using binning technique (BT) method. The figure was developed using the Generic Mapping Tools (GMT, https://www.soest.hawaii.edu/gmt/).

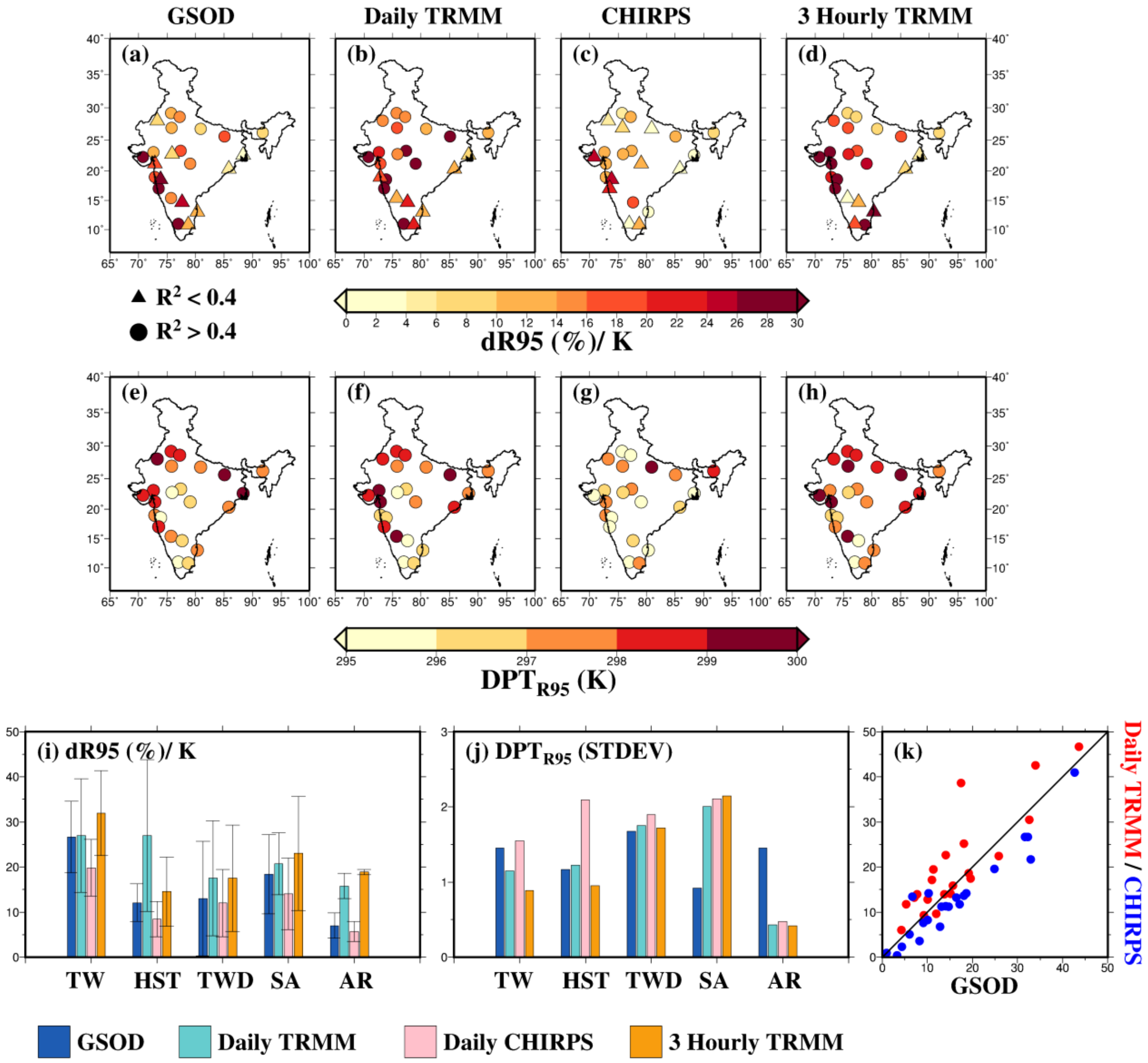

Supplemental Figure S15 (a-d) Regression slopes (dR95/K, %) of extreme rainfall obtained from daily GSOD, daily TRMM, daily CHIRPS and 3-hourly TRMM data, respectively with dewpoint temperature (DPT) for 23 urban areas across India using binning technique (BT), (e-h) peak point temperature (DPT_{R95}) for selected urban areas for same datasets respectively, (i) regression slopes (dR95/K, %) from daily GSOD (blue), daily TRMM (cyan), daily CHIRPS (pink) and 3-hourly TRMM (orange) data for different climatic zones respectively where bars denote mean values and whiskers show standard deviations, (j) same as (i) but for standard deviations (STDEV) in DPT_{R95}, (k) agreement in scaling results between GSOD and Daily TRMM (red) and GSOD and CHIRPS (blue), pooled for all 23 urban areas. The figure was developed using the Generic Mapping Tools (GMT, https://www.soest.hawaii.edu/gmt/).

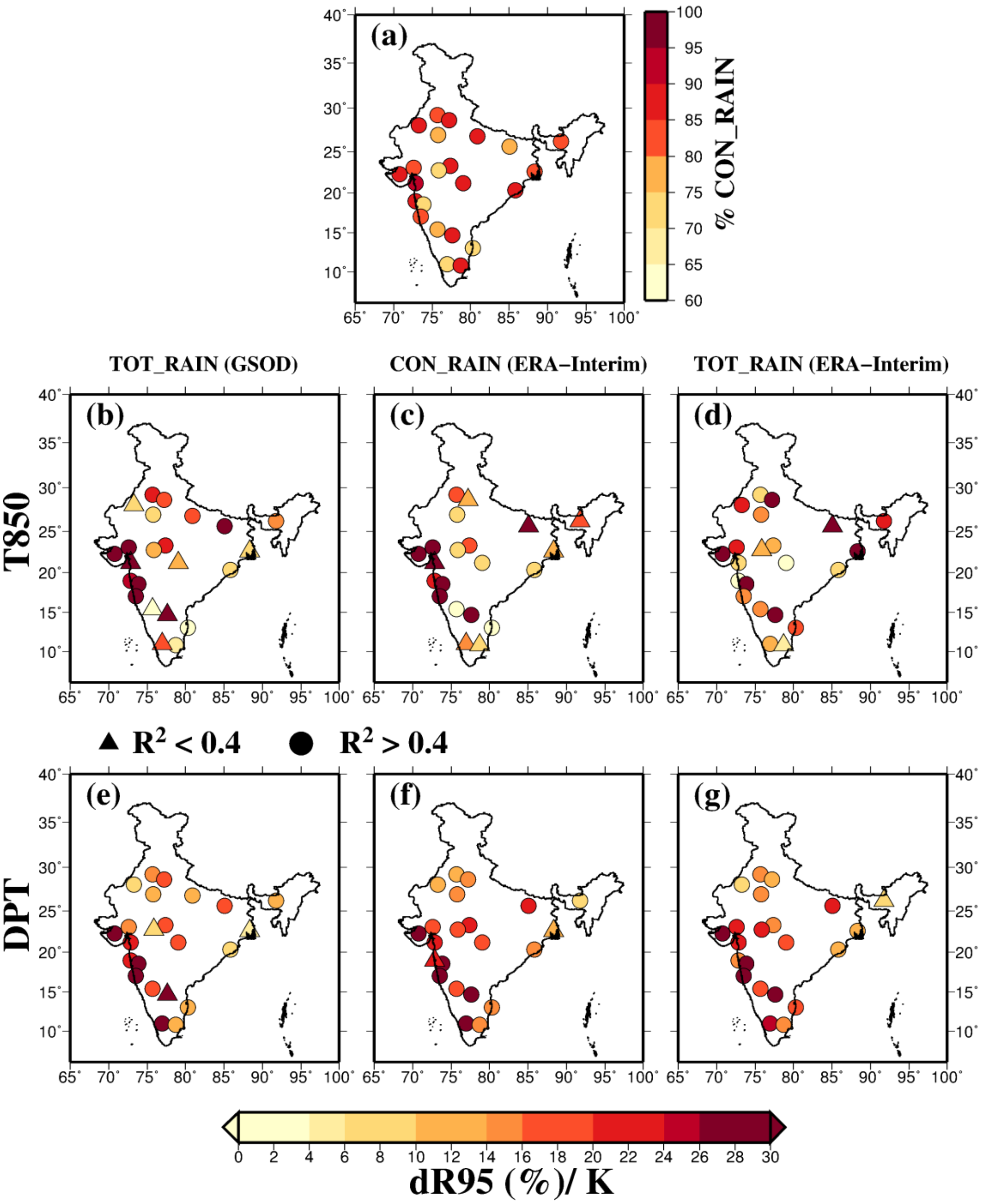

Supplemental Figure S16 (a) Percentage of convective rainfall (CON_RAIN) in total rainfall (TOT_RAIN), obtained from ERA-Interim for the period of 1979-2015 for selected 23 cities across India, (b) regression slopes (dR95/K, %) obtained from daily GSOD considering total rainfall with air temperature at 850 hPa (T850) using quantile regression (QR) method at the 95th percentile, (c) same as (b) but for convective rainfall (CON_RAIN) obtained from ERA-Interim, (d) same as (c) but for total rainfall obtained from ERA-Interim reanalysis product, (e-g) same as (b-d) respectively but for daily dewpoint temperature (DPT). The figure was developed using the Generic Mapping Tools (GMT, https://www.soest.hawaii.edu/gmt/).

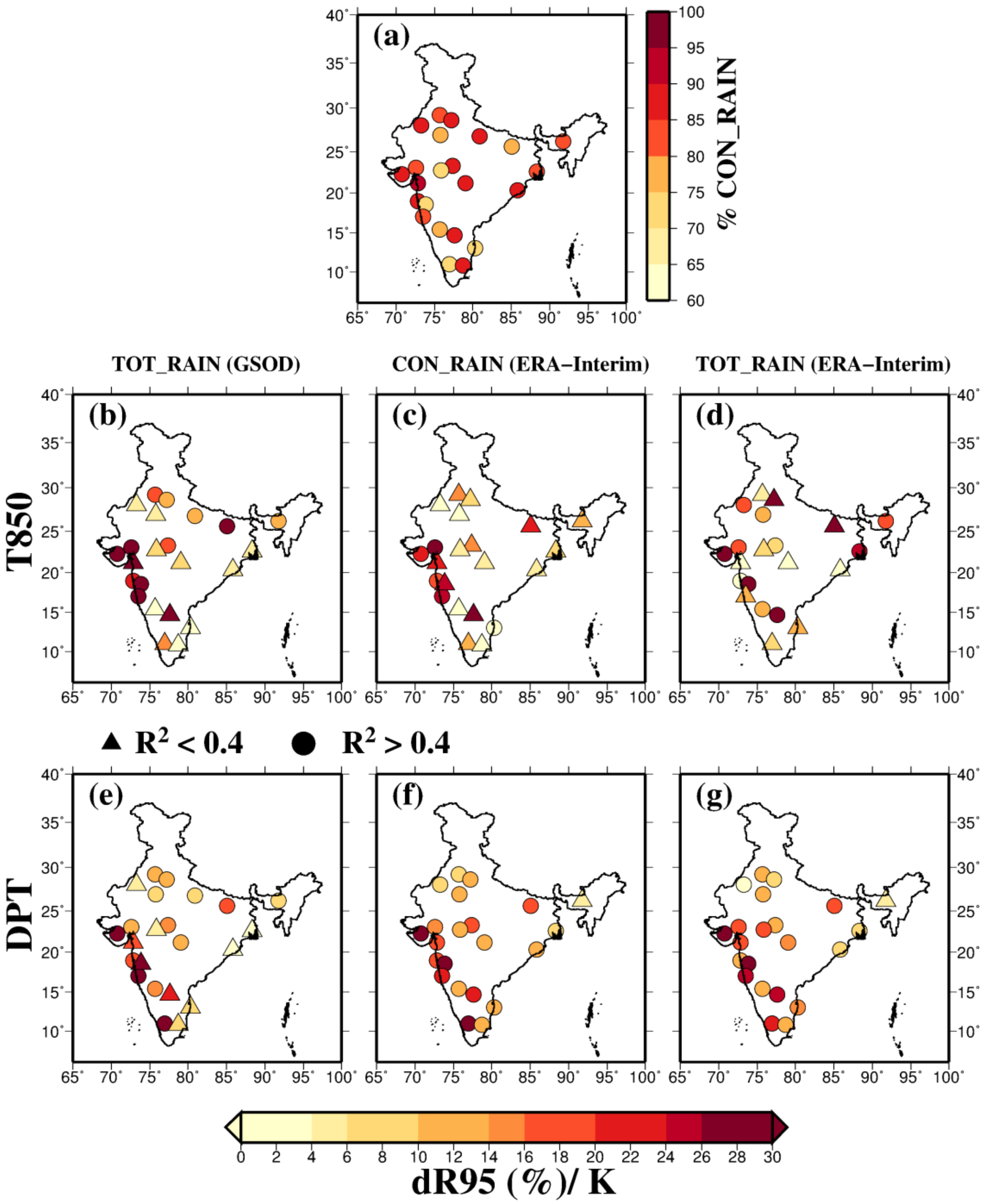

Supplemental Figure S17. Same as Supplemental Figure S16 but using binning technique (BT). The figure was developed using the Generic Mapping Tools (GMT, https://www.soest.hawaii.edu/gmt/).

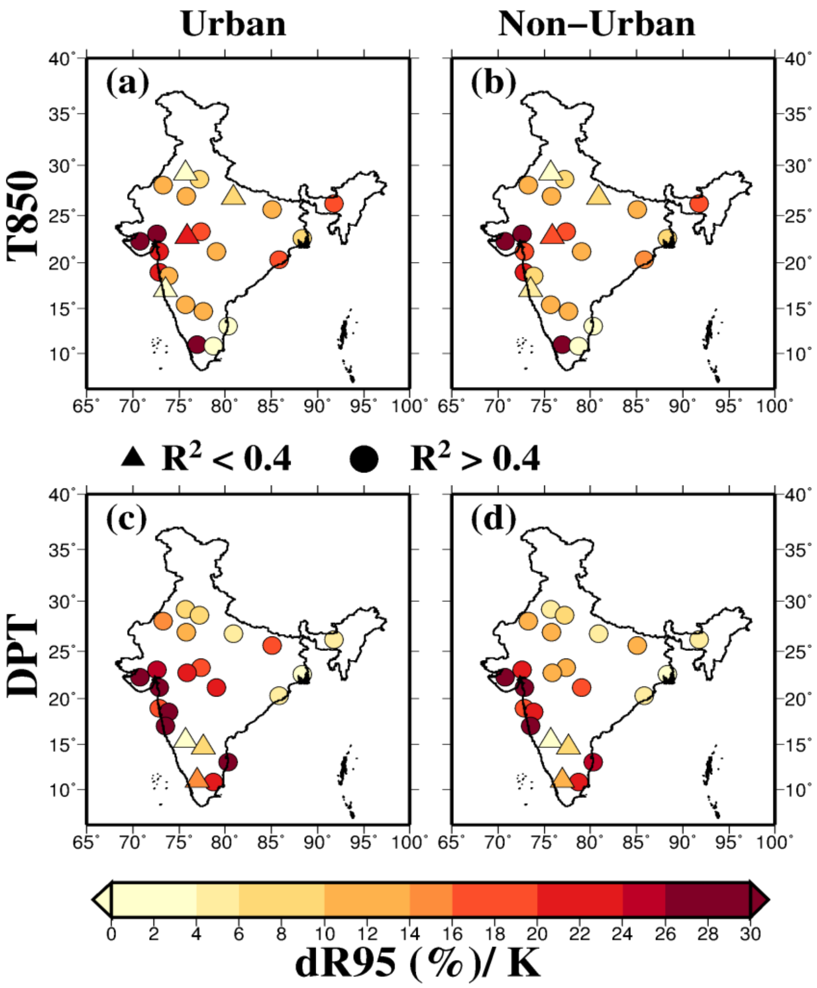

Supplemental Figure18 (a-b) Same as Figure 5 but using binning technique (BT). The figure was developed using the Generic Mapping Tools (GMT, https://www.soest.hawaii.edu/gmt/).

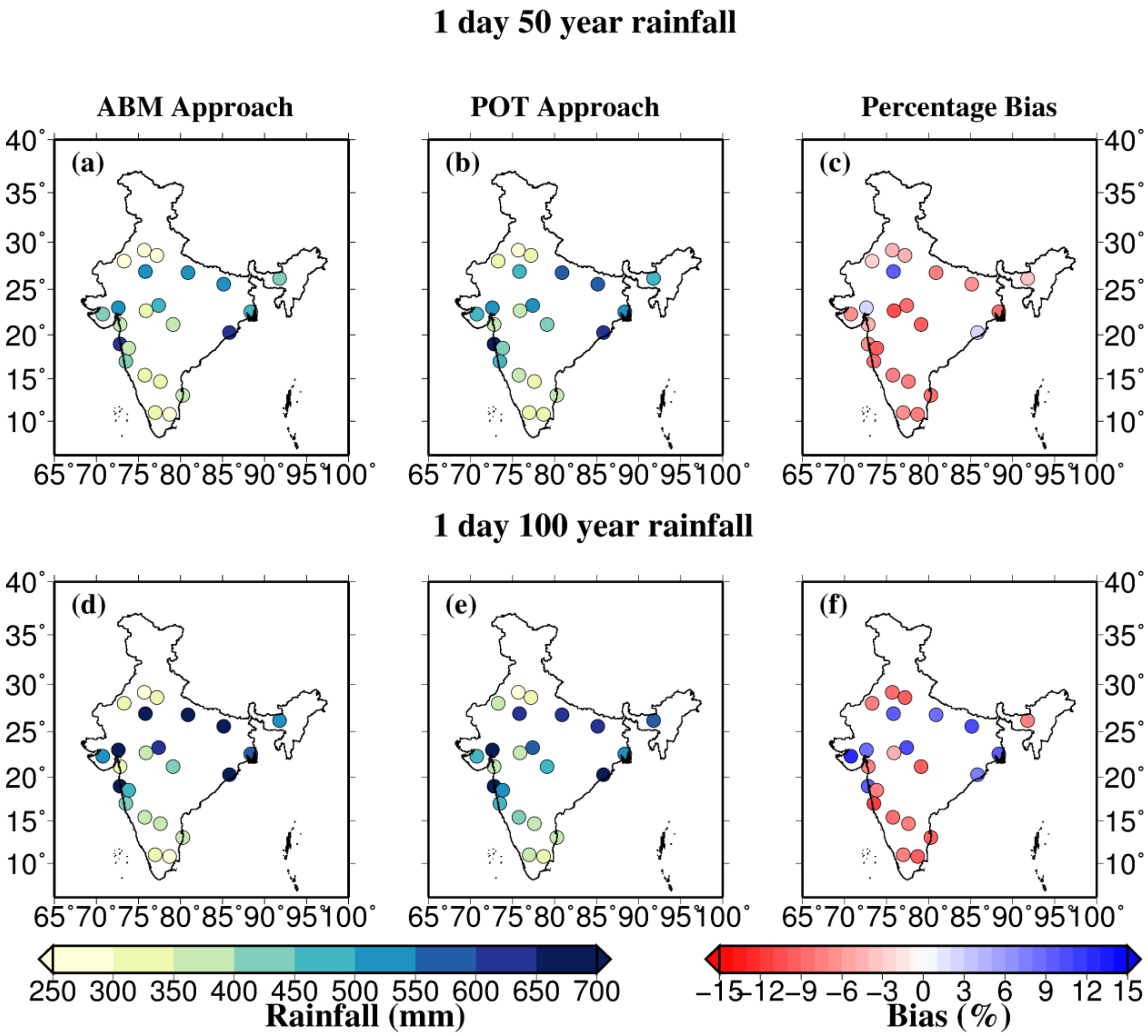

Supplemental Figure S19 (a) 1 day 50 year rainfall maxima (in mm) for 23 cities across India assuming stationary conditions using annual block maxima (ABM) approach, (b) same as (a) but using peak over threshold (POT) approach, (c) percentage bias in 1 day 50 year rainfall maxima using ABM and POT approach considering stationary conditions, (d-f) same as (a-c) respectively but for 1 day 100 year rainfall. The figure was developed using the Generic Mapping Tools (GMT, https://www.soest.hawaii.edu/gmt/).

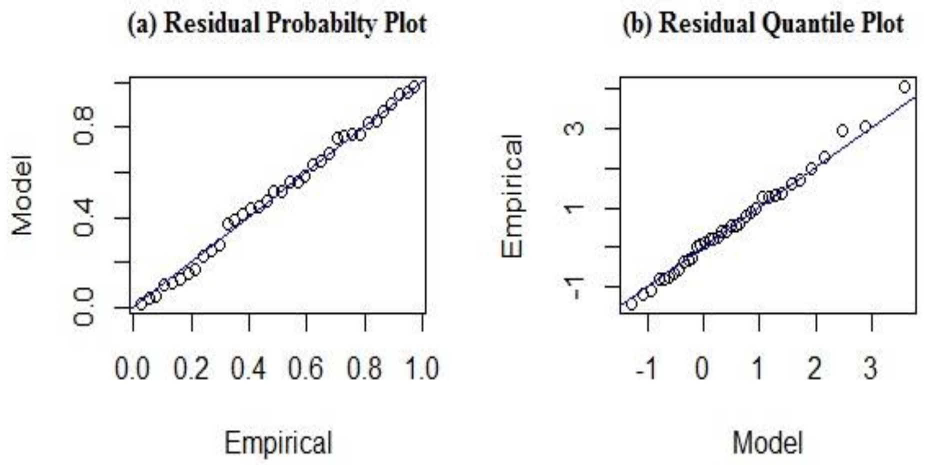

Supplemental Figure S20 (a) Residual Probability Plot for non-stationary GEV model using T850 and DPT as covariates, (b) same as (a) but Residual Quantile Plot. The figure was developed using "gev.diag" function in "ismev" package in statistical programming language 'R'.

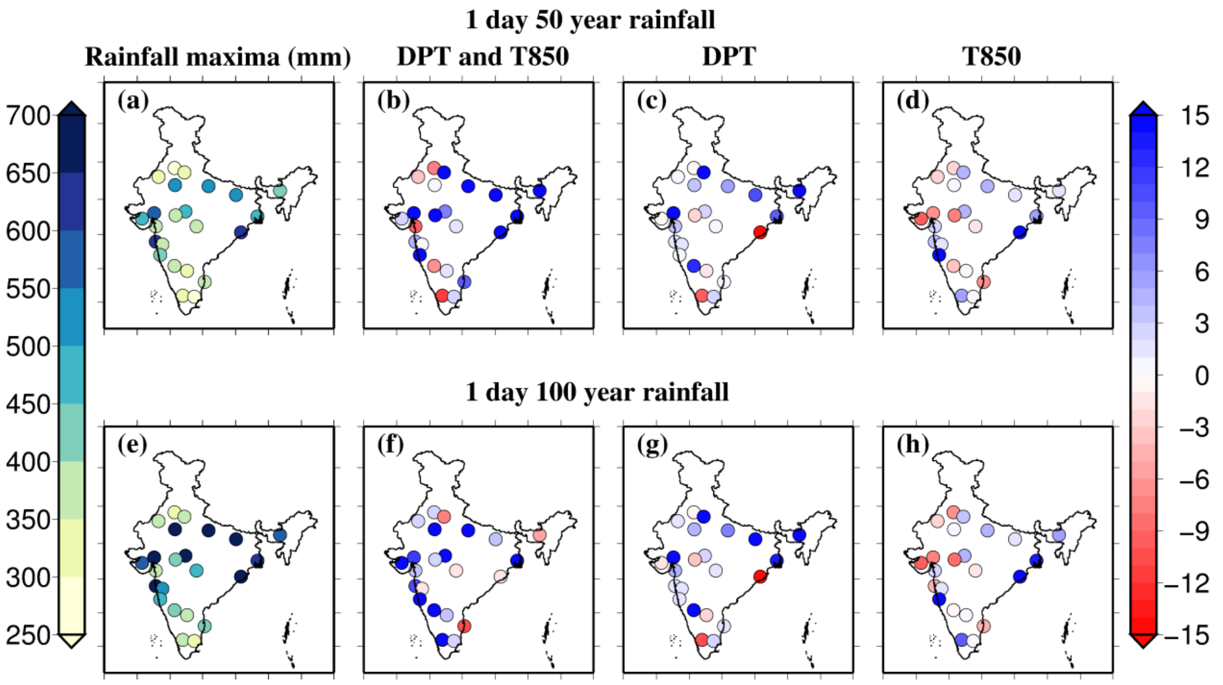

Supplemental Figure S21 (a) 1 day 50 year rainfall maxima (in mm) for 23 urban areas across India assuming stationary conditions, (b) percentage change in 1 day 50 year rainfall maxima considering stationary and nonstationary conditions using DPT and T850 as covariates (c) same as (a) but for 1 day 100 year rainfall maxima, (c) same as (b) but using only DPT as covariate, (d) same as (c) but using T850 as covariate, (e-f) same as (a-d) but for 1 day 100 year rainfall maxima. Return values were estimated using ismev package in "R". The figure was developed using the Generic Mapping Tools (GMT, https://www.soest.hawaii.edu/gmt/).

Supplemental Table S1. Details (location, climate zones and distance from city center) of selected urban areas.

Station id	Name	Longitude	Latitude	Climatic Zone	Distance from city center (km)
1	Ahmedabad	72.58	23.03	SA	7.68
2	Bhopal	77.42	23.25	HST	9.41
3	Bhubaneshwar	85.84	20.27	TWD	3.69
4	Bikaner	73.31	28.02	AR	2.43
5	Mumbai	72.83	18.98	TW	12.72
6	Chennai	80.27	13.08	TWD	13.56
7	Coimbatore	76.97	11.02	TWD	2.24
8	Indore	75.9	22.7	TWD	10.45
9	Nagpur	79.09	21.15	HST	7.84
10	Gadag	75.75	15.4	TWD	12.68
11	Guwahati	91.73	26.18	HST	12.56
12	Hissar	75.7	29.2	SA	5.82
13	Jaipur	75.8	26.9	AR	8.54
14	Lucknow	80.9	26.8	HST	4.472
15	Kolkata	88.37	22.57	TWD	12.32
16	Patna	85.1	25.6	HST	1.56
17	Anantapur	77.6	14.68	SA	11.36
18	Pune	73.86	18.52	TW	9.22
19	Rajkot	70.78	22.3	SA	1.01
20	Ratnagiri	73.5	17	TW	7.37
21	New Delhi	77.21	28.61	SA	2.8
22	Surat	72.83	21.17	TWD	3.35
23	Tiruchirappalli	78.69	10.81	TWD	5.46

SA: Semi Arid

HST: Humid Sub Tropical

TWD: Tropical Wet and Dry

TW: Tropical Wet and Dry

AR: Arid zone

Supplemental Table S2. Root mean square error (RMSE) of different percentile (90th, 95th, 97th, 99th and 99.9^{th}) of rainfall using daily GSOD and daily CHIRPS, after applying different ARFs on gridded CHIRPS data.

Station No.	No ARF	*arf1*	*arf2*	*arf3*	*arf4*	*arf5*
1	121.09	78.90	120.33	131.66	90.12	115.87
2	92.40	51.83	61.83	115.71	91.65	87.29
3	113.31	67.16	81.76	101.99	112.58	108.26
4	140.75	121.57	128.86	98.76	140.49	138.95
5	82.31	196.63	126.12	208.45	81.54	78.65
6	56.43	89.23	46.34	285.13	55.13	47.80
7	78.98	44.39	57.25	41.71	78.51	75.70
8	68.87	37.02	38.29	130.14	68.07	63.44
9	97.00	64.53	75.07	78.50	96.49	93.49
10	74.47	13.16	29.25	126.75	73.49	67.68
11	43.05	43.47	23.86	154.55	42.25	37.63
12	60.95	19.50	31.53	83.93	60.29	56.39
13	131.30	102.30	112.55	89.39	130.87	128.36
14	148.83	114.01	125.83	209.33	148.30	145.16
15	100.96	55.10	67.59	117.14	100.16	95.49
16	148.56	112.79	125.72	192.32	148.04	145.02
17	113.71	93.38	101.02	72.23	113.43	111.78
18	148.64	122.71	131.09	123.18	148.22	145.76
19	121.21	71.57	86.72	116.42	120.40	115.64
20	55.10	102.24	197.87	219.97	57.08	71.13
21	63.36	38.11	43.60	83.41	62.86	59.97
22	74.73	84.62	55.98	258.40	73.66	67.65
23	52.12	15.18	18.36	105.32	51.36	46.90

Supplemental Table S3. Deviance Statistic (D) test to evaluate improvement in the non-stationary GEV model over stationary GEV model.

S.No	Station	Negative log-likelihood (nlh)		Deviance Statistic(D)
		Stationary	Non-stationary	
1	Ahmedabad	190.19	187.88	4.62
2	Bhopal	183.51	181.47	4.08
3	Bhubaneshwar	193.77	191.23	5.08
4	Bikaner	173.43	171.24	4.39
5	Mumbai	188.54	186.47	4.15
6	Chennai	188.33	186.22	4.23
7	Coimbatore	176.69	173.27	6.84
8	Indore	184.19	182.05	4.27
9	Nagpur	182.31	180.04	4.54
10	Gadag	183.98	182.02	3.91
11	Guwahati	181.06	179.08	3.96
12	Hissar	178.09	173.23	9.72
13	Jaipur	180.56	178.52	4.08
14	Lucknow	188.03	184.31	7.44
15	Kolkata	190.77	188.45	4.65
16	Patna	182.92	181.00	3.85
17	Anantapur	176.56	174.49	4.12
18	Pune	176.76	174.71	4.10
19	Rajkot	192.52	190.30	4.43
20	Ratnagiri	185.69	183.04	5.30
21	New Delhi	176.04	174.04	4.00
22	Surat	194.72	190.38	8.68
23	Tiruchirappalli	179.44	177.43	4.03

Supplemental Table S4. PSR test to examine Non-Stationarity.

S.No	Station	p-value for T	Non stationarity
1	Ahmedabad	0	Yes
2	Bhopal	0	Yes
3	Bhubaneshwar	0	Yes
4	Bikaner	0	Yes
5	Mumbai	0	Yes
6	Chennai	0	Yes
7	Coimbatore	0	Yes
8	Indore	0	Yes
9	Nagpur	0	Yes
10	Gadag	0	Yes
11	Guwahati	0	Yes
12	Hissar	0	Yes
13	Jaipur	0	Yes
14	Lucknow	0	Yes
15	Kolkata	0	Yes
16	Patna	0	Yes
17	Anantapur	0	Yes
18	Pune	0	Yes
19	Rajkot	0	Yes
20	Ratnagiri	0	Yes
21	New Delhi	0	Yes
22	Surat	0	Yes
23	Tiruchirappalli	0	Yes

References

1. Asquith, W. H. Areal-reduction factors for the precipitation of the 1-day design storm in Texas. US Geological Survey, WaterResources Investigations Report 99-4267, Austin, Tex., 81 (1999).

2. Allen, Robert J., and Arthur T. DeGaetano. "Areal reduction factors for two eastern United States regions with high rain-gauge density." Journal of Hydrologic Engineering **10**, no. 4, 327-335 (2005)

3. Olivera, Francisco, et al. "Estimation of average rainfall areal reduction factors in Texas using NEXRAD data." Journal of Hydrologic Engineering **13**, no. 6, 438-448 (2008).

4. Svensson, Cecilia, and David A. Jones. "Review of methods for deriving areal reduction factors." Journal of Flood Risk Management **3**, no. 3, 232-245 (2010).

5. Mishra, Vimal, Francina Dominguez, and Dennis P. Lettenmaier. "Urban precipitation extremes: How reliable are regional climate models?." Geophysical Research Letters **39**, no. 3 (2012).

6. Tripathi, Om P., and Francina Dominguez. "Effects of spatial resolution in the simulation of daily and subdaily precipitation in the southwestern US." Journal of Geophysical Research: Atmospheres **118,** no. 14, 7591-7605 (2013).

7. Lincoln, W. Scott, Katie E. Landry-Guyton, and David S. Schlotzhauer. "Creation of Rainfall Areal Reduction Factors from the Basin-Averaged Rainfall Record at the Lower Mississippi River Forecast Center", LMRFC Technical Report , National Weather Service (2016).

8. Mishra, V., Wallace, J. M. & Lettenmaier, D. P. Relationship between hourly extreme precipitation and local air temperature in the United States: Extreme Precipitation And Temperature. Geophys. Res. Lett. **39,** no. 16 (2012).

9. Kunkel, Kenneth E., David R. Easterling, David AR Kristovich, Byron Gleason, Leslie Stoecker, and Rebecca Smith. "Meteorological causes of the secular variations in observed extreme precipitation events for the conterminous United States." Journal of Hydrometeorology . **3,** 1131-1141 (2012).
